# Sub-organellar mitochondrial hydrogen peroxide observed using a SNAP tag targeted coumarin-based fluorescent reporter^☆^

**DOI:** 10.1016/j.redox.2025.103502

**Published:** 2025-01-20

**Authors:** Ross Eaglesfield, Erika Fernandez-Vizarra, Erik Lacko, Stuart T. Caldwell, Nikki L. Sloan, Daniel Siciarz, Richard C. Hartley, Kostas Tokatlidis

**Affiliations:** a School of Molecular Biosciences, University of Glasgow, G12 8QQ, UK; b School of Chemistry, University of Glasgow, G12 8QQ, UK; c National Renewable Energy Laboratory, Golden, CO, USA; d Department of Biochemistry and Molecular and Cellular Biology, Faculty of Health and Sport Sciences, University of Zaragoza, 22002, Spain

**Keywords:** Mitochondria, ROS, Self-labeling proteins, Redox signalling, Oxidative stress, Sub-cellular compartments

## Abstract

Mitochondria are major sites of reactive oxygen species (ROS) production within cells. ROS are important signalling molecules, but excessive production can cause cellular damage and dysfunction. It is therefore crucial to accurately determine when, how and where ROS are produced within mitochondria. Previously, ROS detection involved various chemical probes and fluorescent proteins. These have limitations due to accumulation of the molecules only in the mitochondrial matrix, or the need for a new protein to be expressed for every different species. We report dynamic H_2_O_2_ flux changes within all mitochondrial sub-compartments with striking spatial resolution. We combined specific targeting of self-labeling proteins with novel H_2_O_2_-reactive probes. The approach is broad-ranging and flexible, with the same expressed proteins loadable with different dyes and sensors. It provides a framework for concomitant analysis of other chemical species, beyond ROS, whose dynamics within mitochondria are yet unknown, without needing to engineer new proteins.

## Highlights

• SNAP tags can be used to localize a new PeroxyCoum H_2_O_2_-sensor to five distinct mitochondrial sub-compartments in addition to the cytosol.
• PeroxyCoum detects dynamic flux changes of H_2_O_2_ in mitochondria with unprecedented sub-organellar spatial resolution.
• Dual labelling with both SNAP and Halo tags paves the way for multifaceted detection of small chemical species *in vivo.*

## 1 Introduction

Reactive oxygen species (ROS) are often thought of as toxic compounds that result in oxidative stress conditions which manifest in many human disease states, especially age-related conditions [1,2]. Some ROS, typically hydrogen peroxide (H_2_O_2_), also act as important regulators of critical cellular functions ranging from apoptosis to cell growth and proliferation, and they are involved in a wide range of signalling processes [[3], [4], [5]]. This regulatory role critically depends on the location and concentration of H_2_O_2_ [6]. It is therefore crucial for cells to regulate the production, accumulation, and trafficking of a variety of ROS in order to maintain balance without leading to detrimental oxidative stress conditions.

Mitochondria are one of the major sites of ROS production in cells, mainly due to the presence of the respiratory electron transport chain at the inner mitochondrial membrane (IMM). The production of ROS is primarily due to leakage during the electron transfer events that occur during respiration [7]. Some mitochondrially-produced ROS, such as superoxide O_2_^•-^, are highly reactive and damaging. As such, cells utilize scavenging enzymes like the superoxide dismutases (SODs) to rapidly turn O_2_^•-^ into the less reactive H_2_O_2_ [8]. Full reduction of molecular oxygen to water is well controlled in complex IV. However, leakage of electrons from complexes I and III of the electron transport chain can lead to partial reduction of molecular oxygen to O_2_^•-^. For example, the Q_o_ site of complex III is a major site of O_2_^•-^ production due to the direct reaction of ubisemiquinone with molecular oxygen. The resulting O_2_^•-^ is released into both the matrix and the intermembrane space (IMS) in an approximately 1:1 ratio [9]. In addition, under the conditions of a highly reduced coenzyme Q pool and high membrane potential, reverse electron transport (RET) occurs and then the major site of partial molecular oxygen reduction is the FMN site of complex I [10,11]. In this case, the release of O_2_^•-^ is only to the matrix, where it can directly damage key enzymes containing metal clusters, such as aconitase [12]. O_2_^•-^ has a pK_aH_ of 4.7 and the protonated hydroperoxyl radical HO_2_^•^ can penetrate the membranes and damage lipids through initiation of a radical chain reaction by abstracting the bis-allylic hydrogen atoms of polyunsaturated fatty acid chains [13]. Furthermore, in conjunction with H_2_O_2_, O_2_^•-^ can sustain the iron-catalyzed Haber Weiss reaction which generates the damaging hydroxyl radical, which can induce ferroptosis [14]. To mitigate the potential damage created by O_2_^•-^, it is rapidly dismutated into H_2_O_2_ and molecular oxygen by either SOD1 in the IMS or SOD2 in the matrix. H_2_O_2_ is much less reactive than O_2_^•-^ and as such it is thought to freely diffuse from the IMS to the cytosol through the VDAC/porin proteins of the outer mitochondrial membrane (OMM). The movement of H_2_O_2_ across the inner membrane is not fully understood. It is possible that the molecule freely diffuses through the lipid bilayer, however it is also hypothesized that an as yet unidentified transport protein exists to facilitate its movement [15]. The cytosol, IMS and matrix all contain antioxidant scavenging proteins that remove H_2_O_2_ from the local environment, which leads to large gradients of H_2_O_2_ concentration within these sub-compartments [16].

The production and, particularly, the flux of H_2_O_2_ across mitochondrial sub-compartments bear significant and wide-ranging implications for mammalian cell homeostasis and human health. However, understanding of this process remains relatively limited. Studies have generally relied on chemical probes that require the mitochondrial inner membrane electrochemical potential in order to localize correctly (for example MitoB [17]), or on large fluorescent protein-based probes (like HyPer and roGFP) [15,18,19]. MitoB has the advantage that it can be used in any cell type and in whole organisms [20], but its mechanism of accumulation only allows it to detect bulk H_2_O_2_ concentrations in the matrix of active mitochondria. Fluorescent protein-based probes (e.g. HyPer and roGFP) have the advantage that they can be expressed in the cells with targeting sequences or as fusion proteins to allow localization to any cellular compartment. Nonetheless, the alteration of photophysical properties or the detection of different species requires the expression of distinct proteins. Finally, in some systems, the read out might be influenced by the activity of reducing enzymes [15,18,19]. A recent example of this highly targeted approach in living cells successfully utilized a fluorescent protein to analyze H_2_O_2_ dynamics and release from mitochondria with sub-mitochondrial spatial resolution [15,18].

Here we present an alternative strategy. We thought that self-labeling proteins would provide a more versatile approach to investigating sub-mitochondrial redox species and might allow even higher resolution, together with multiplexing. Self-labeling proteins [21], such as SNAP-tag, HaloTag, and CLIP-tag, will attach any type of small molecule sensor to themselves, provided the small molecule bears the tag specifically recognized by the protein. This allows the synthetic small molecule sensor to be optimized for the correct reactivity and photophysical properties separately from the self-labeling protein. Furthermore, a cell line expressing a self-labeling protein can be used to detect different species at will, by using different small molecule sensors. The range of self-labeling proteins also holds out the possibility of multiplexing with different sensors loaded onto each type of protein using the different specificity of tag recognition. Previously, SNAP-tags have been successfully used to target various intracellular organelles for the detection of a number of species including metal ions and H_2_O_2_ [[22], [23], [24]]. Therefore, we decided to use the precise targeting enabled by SNAP-tag to localize small molecule fluorescent sensors and so achieve sub-organellar detection of H_2_O_2_ within mitochondria. The sensors would be irreversibly converted by hydrogen peroxide into a non-redox active fluorescent dye with the read out integrated over time to limit the effect of endogenous reducing enzymes.

In this work, we have been able to determine precise, dynamic changes in H_2_O_2_ in response to metabolic alterations induced by different nutrients and with hitherto unprecedented submitochondrial spatial resolution. This is an important step forward to understand how cells respond to stress and metabolic changes in a compartment-specific manner to ensure survival. To this end we harnessed a localized detection of H_2_O_2_ using specifically-targeted SNAP-tags. Additionally, we capitalized on this approach via the synthesis and characterization of a new, coumarin-based, small chemical probe for the detection of H_2_O_2_. Thus, we have been able to achieve the precise localization of the tag to the cytosol as well as to all mitochondrial sub-compartments, i.e., the OMM, the IMS, the cristae lumen, and the matrix. In addition, this allowed us to localize our reporter specifically to different sites within the IMS, allowing the determination of H_2_O_2_ gradients even within a specific sub-compartment. We show that a chloropyrimidine coumarin probe can be used, in conjunction with SNAP tagged mitochondrial proteins, to detect H_2_O_2_ with sub-organellar resolution upon inducing oxidative stress. Furthermore, we used these probes to gain insight into changes in H_2_O_2_ production in relation to alterations in cellular metabolism in cells grown in galactose-based media (leading to increased respiratory conditions) compared to those grown in glucose (i.e., in more glycolytic conditions), finding increased H_2_O_2_ flux in conditions favoring respiration. Finally, we expanded our approach to show dual targeting of two spectrally distinct chemical probes to multiple subcellular locations by the combined use of proteins containing the SNAP-tag with others containing the HaloTag, a differently reactive self-labelling tag [25]. This has also provided a proof of concept and framework for further studies, not only for ROS detection, but also for any ion or molecule detectable by a small molecule probe. The approach taken here has allowed us to measure ROS dynamics within mitochondrial sub-compartments of living cells with striking spatial resolution, as well as provide a framework for the analysis of other chemical species whose dynamics within mitochondria are as yet unknown.

## 2 Methods

### 2.1 Chemical synthesis

**Fig. 1:**
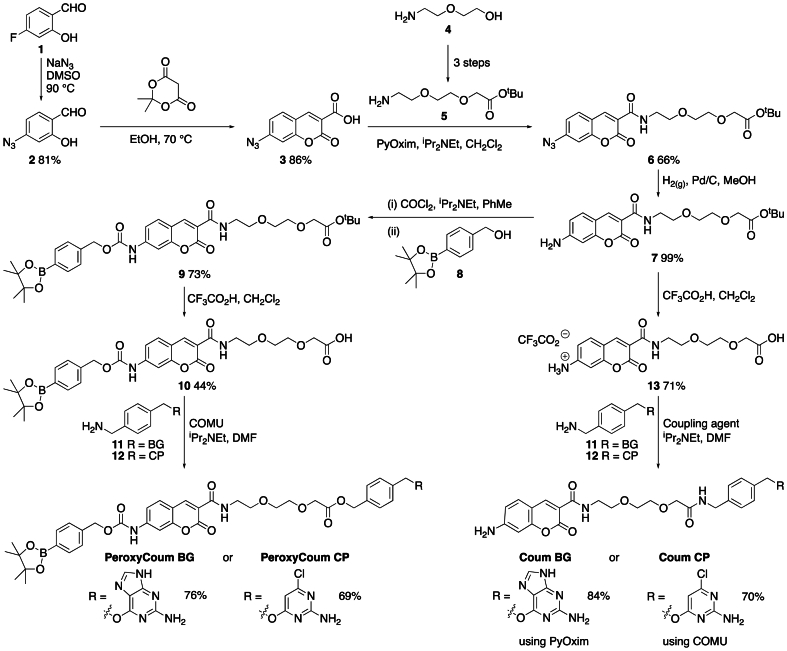
Synthesis of molecular probes PeroxyCoum BG, PeroxyCoum CP, Coum BG and Coum CP used in this study.

PeroxyCoum BG, PeroxyCoum CP, Coum BG and CoumCP were synthesized in seven steps from 4-fluoro-2-hydroxybenzaldehyde **1** (Fig. 1). Azidocoumarin **3** was synthesized in good yield from 4-fluoro-2-hydroxybenzaldehyde **1**. The fluoride was displaced by S_N_Ar to give azidosalicylaldehyde **2** which underwent Knovenagel condensation with Meldrums acid to give the coumarin carboxylic acid **3**. Acid **3** was coupled with known amine **5** [26] to give amide **6** which was subsequently reduced to give amine **7**. Conversion of the amine to the isocyanate then trapping with benzylic alcohol **8** gave carbamate **9** in good yield. Deprotection of the *tert*-butyl ester using TFA gave the free carboxylic acid **10** in modest yield. Amide coupling with commercially available O^6^-[4-(aminomethyl)benzyl]guanine **11** or 4-[(4’-(aminomethyl)benzyloxy]-6-chloropyrimidine-2-amine **12** (synthesis reported by Srikun et al.) [22] gave PeroxyCoum BG or PeroxyCoum CP respectively. The *tert*-Butyl ester of intermediate **7** was cleaved with acid and the resulting carboxylic acid **13** coupled with amines **11** and **12** to give Coum BG and Coum CP, respectively. Details of synthetic protocols are found below. The assignments of signals in the ^1^H NMR spectra use the numbering/lettering shown in Fig. S1.

Synthesis of 2-hydroxy-4-azidobenzaldehyde**2**. Sodium azide (1.64 g, 27.1 mmol, 1.2 eq) was added to a solution of 2-hydroxy-4-fluorobenzaldehyde **1** (3.16 g, 22.6 mmol, 1.0 eq) in degassed DMSO (30 mL). The solution was heated to 90 °C under an atmosphere of argon overnight. After cooling to RT the solution was poured into water (200 mL) and extracted into EtOAc (50 mL). The aqueous layer was reextracted with EtOAc (50 mL) and the combined organics washed with brine (3 × 200 mL), dried over magnesium sulfate and concentrated under vacuum to give the aldehyde **2** as a beige solid (3.00 g, 81 %). ^1^H NMR data are in agreement with literature [27].

Synthesis of 7-azidocoumarin-3-carboxylic acid**3**. Piperidine (142 μL, 1.44 mmol, 0.05 eq) was added to a solution of Meldrum's acid (5.01 g, 34.8 mmol, 1.2 eq) and 2-hydroxy-4-azidobenzaldehyde **2** (4.73 g, 29.0 mmol, 1.0 eq) in EtOH (50 mL). The solution was heated under an atmosphere of argon at 70 °C for 2 h. The solution was cooled to RT and the resulting solid filtered, washed with EtOH (50 mL) and dried under vacuum to give the coumarin **3** as a beige solid (5.80 g, 86 %). ^1^H NMR data are in agreement with literature [28].

Synthesis of*tert*-butyl 2-(2'-(2″-aminoethoxy)ethoxy)acetate**5**. Amine **5** was synthesized on a 20 mmol scale according to the method of Kohl et al. [26].

Synthesis of azidocoumarin**6**. PyOxim (675 mg, 1.28 mmol, 1.05 eq) was added to a solution of carboxylic acid **3** (282 mg, 1.22 mmol, 1.0 eq), amine **5** (268 mg, 1.22 mmol, 1.0 eq) and diisopropylethylamine (432 μL, 0.2.44 mmol, 2.0 eq) in dry CH_2_Cl_2_ (15 mL). The solution was stirred at RT overnight under an atmosphere of argon. The solution was then diluted with CH_2_Cl_2_ (30 mL), washed with 1 M hydrochloric acid (2 × 50 mL), dried over magnesium sulfate and concentrated under vacuum. The residue was purified by column chromatography using a 12 g Agela cartridge eluting with EtOAc:Hexanes (30:70 increasing to 90:10 over 10 column volumes) to give the amide **6** as a yellow solid (350 mg, 66 %). *δ*_H_ (400 MHz: CDCl_3_): 1.45 (9H, s, CH_3_), 3.66–3.73 (8H, m, 4 × OCH_2_), 4.03 (2H, s, CH_2_), 7.00–7.02 (2H, m, *J* = 8.7, 7.3 Hz, H-6 + 8), 7.64 (1H, d, *J* = 8.1 Hz, H-5), 8.82 (1H, s, H-4), 8.94 (1H, broad s, NH).*δ*_C_ (101 MHz: CDCl_3_): 28.21 (CH_3_), 39.81 (CH_2_), 69.22 (CH_2_), 69.74 (CH_2_), 70.58 (CH_2_), 70.86 (CH_2_), 81.67 (C), 106.73 (CH), 115.77 (C), 116.65 (CH), 117.26 (C), 131.34 (CH), 146.48 (C), 147.59 (CH), 155.75 (C), 160.95 (C), 161.70 (C), 169.82 (C). m/z (ESI): Found: 455.1526. C_20_H_24_O_7_N_4_Na requires (*M + Na)*^*+*^, 455.1537.

Synthesis of aminocoumarin**7**. Azide **6** (200 mg, 0.46 mmol, 1.0 eq) and Pd/C (30 mg) in MeOH (30 mL) were hydrogenated under a balloon of hydrogen for 3h. The solution was filtered through Celite and the Celite washed with MeOH (30 mL). The MeOH was concentrated under vacuum to give the amine **7** as a bright yellow solid (186 mg, 99 %). *δ*_H_ (400 MHz: CDCl_3_): 1.43 (9H, s, CH_3_), 3.59–3.73 (8H, m, 4 × OCH_2_), 4.01 (2H, s, CH_2_), 5.01 (2H broad s, NH_2_), 6.55–6.58 (2H, m, H-6 + 8), 7.29 (1H, d, *J* = 8.3 Hz, H-5), 8.58 (1H, s, H-4), 8.97 (1H, t, *J* = 5.2 Hz, NH).*δ*_C_ (101 MHz: CDCl_3_): 28.15 (CH_3_), 39.54 (CH_2_), 69.07 (CH_2_), 69.85 (CH_2_), 70.41 (CH_2_), 70.75 (CH_2_), 81.74 (C), 99.55 (CH), 109.86 (C), 111.41 (C), 113.04 (CH), 131.38 (CH), 148.28 (CH), 153.74 (C), 157.27 (C), 162.37 (C), 163.05 (C), 169.80 (C). m/z (ESI): Found: 429.1632. C_20_H_26_O_7_N_2_Na requires (*M + Na)*^*+*^, 429.1631.

Synthesis of carbamate**9**. Phosgene (15 % wt in toluene) (130 μL, 0.17 mmol, 1.0 eq) was added to a solution of amine **7** (69 mg, 0.17 mmol, 1.0 eq) and diisopropylethylamine (58 μL, 0.34 mmol, 2.0 eq) in dry toluene (3 mL) under an atmosphere of argon. The solution was heated to 90 °C for 45 min, cooled to RT then 4-(hydroxymethyl)phenylboronic acid pinacol ester **8** (60 mg, 0.25 mmol, 1.5 eq) added. The solution was heated to 90 °C for a further hour. The solution as cooled to R.T, concentrated under vacuum and purified by column chromatography using a 12 g Agela cartridge eluting with CH_2_Cl_2_:EtOAc (100:0 increasing to 40:60 over 10 column volumes) to give the carbamate **9** as an off white solid (83 mg, 73 %). *δ*_H_ (400 MHz: CDCl_3_): 1.34 (12H, s, CH_3_), 1.46 (9H, s, CH_3_), 3.63–3.68 (4H, m, NCH_2_ + OCH_2_), 3.68–3.70 (2H, m, OCH_2_), 3.73–3.75 (2H, m, OCH_2_), 4.04 (2H, s, CH_2_), 5.24 (2H, s, ArC*H*_2_), 7.31 (1H, dd, *J* = 8.6, 2.0 Hz, H-6), 7.34 (1H, s, NH), 7.39 (2H, d, *J* = 8.0 Hz, H_A_ or H_B_), 7.56 (1H, d, *J* = 8.6, H-5), 7.67 (1H, d, *J* = 2.0 Hz, H-8), 7.81 (2H, d, *J* = 8.0 Hz, H_A_ or H_B_), 8.77 (1H, s, H-4), 9.00 (1H, s, NH). *δ*_C_ (101 MHz: CDCl_3_): 24.99 (CH_3_), 28.24 (CH_3_), 39.79 (CH_2_), 67.66 (CH_2_), 69.26 (CH_2_), 69.78 (CH_2_), 70.63 (CH_2_), 70.90 (CH_2_), 81.70 (C), 84.08 (C), 105.28 (CH), 114.20 (C), 115.60 (CH), 116.19 (C), 127.59 (CH), 130.70 (CH), 135.26 (CH), 138.44 (C), 143.77 (C), 147.86 (CH), 152.65 (C), 155.90 (C), 161.56 (C), 162.13 (C), 169.86 (C). Note – C–B carbon not observed. m/z (ESI): Found: 689.2830. C_34_H_43_BO_11_N_2_Na requires (*M + Na)*^*+*^, 689.2852.

Synthesis of acid**10**. Trifluoroacetic acid (500 μL) was added to a solution of ester **9** (70 mg, 0.10 mmol, 1.0 eq) in dry CH_2_Cl_2_ (3 mL) under an atmosphere of argon. The solution was stirred at RT for 2 h, diluted with CH_2_Cl_2_ (10 mL), washed with brine (2 × 40 mL), dried over magnesium sulfate and concentrated under vacuum to give the carboxylic acid **10** as a white solid (28 mg, 44 %). *δ*_H_ (400 MHz: CDCl_3_): 1.34 (12H, s, CH_3_), 3.63–3.68 (4H, m, NCH_2_ + OCH_2_), 3.69–3.71 (2H, m, OCH_2_), 3.75–3.78 (2H, m, OCH_2_), 4.17 (2H, s, CH_2_), 5.24 (2H, s, ArC*H*_2_), 7.35 (1H, dd, *J* = 8.6, 1.9 Hz, H-6), 7.38 (2H, d, *J* = 7.9 Hz, H_A_ or H_B_), 7.56 (1H, d, *J* = 8.6, H-5), 7.61 (1H, s, NH), 7.63 (1H, d, *J* = 1.9 Hz, H-8), 7.81 (2H, d, *J* = 7.9 Hz, H_A_ or H_B_), 8.78 (1H, s, H-4), 9.08 (1H, t, *J* = 4.6 Hz, NH). *δ*_C_ (101 MHz: CDCl_3_): 24.99 (CH_3_), 39.57 (CH_2_), 67.66 (CH_2_), 68.89 (CH_2_), 69.81 (CH_2_), 70.33 (CH_2_), 71.30 (CH_2_), 84.11 (C), 105.28 (CH), 114.11 (C), 115.72 (CH), 115.77 (C), 127.58 (CH), 130.85 (CH), 135.25 (CH), 138.48 (C), 144.09 (C), 148.30 (CH), 152.79 (C), 155.83 (C), 161.80 (C), 162.49 (C), 172.22 (C). Note – C–B carbon not observed. m/z (ESI): Found: 633.2212. C_30_H_35_BO_11_N_2_Na requires (*M + Na)*^*+*^, 633.2212.

Synthesis of acid**13**. TFA (1 mL) was added to a solution of ^*t*^butyl ester **7** (110 mg, 0.27 mmol, 1.0 eq) in CH_2_Cl_2_(4 mL). The solution was stirred at R.T. for 2h then concentrated under vacuum. The product was redissolved in CH_2_Cl_2_and concentrated under vacuum three times. Diethyl ether was added, the resulting solid filtered and washed with ether to give the acid as a yellow solid (86.0 mg, 71 %) which was used without further purification or analysis.

Synthesis of**PeroxyCoum BG**. COMU (47 mg, 0.110 mmol, 1.5 eq) was added to a suspension of 6-[4'-(aminomethyl)benzyloxy]-7H-purin-2-amine **11** (30 mg, 0.110 mmol, 1.5 eq), carboxylic acid **10** (45 mg, 0.074 mmol, 1.0 eq) and diisopropylethylamine (40 μL, 0.222 mmol, 3.0 eq) in dry DMF (3 mL). The resulting solution was stirred overnight, concentrated under vacuum and purified by column chromatography using a 12 g Agela cartridge eluting with CH_2_Cl_2_:MeOH (100:0 increasing to 80:20 over 10 column volumes) to give the amide as a beige solid (48.3 mg, 76 %). *δ*_H_ (400 MHz, DMSO‑*d*_6_): 1.29 (12H, s, 4 × CH_3_), 3.47 (2H, apparent q, *J* = 5.5 Hz, NH*CH*_*2*_CH_2_), 3.55 (2H, t, *J* = 5.5 Hz, NHCH_2_*CH*_*2*_), 3.56–3.67 (4H, q, *J* = 1.2 Hz, 2 × OCH_2_), 3.95 (2H, s, CH_2_), 4.32 (2H, d, *J* = 6.2 Hz, Ar*CH*_*2*_NH), 5.24 (2H, s, CH_2_), 5.42 (2H, s, CH_2_), 6.26 (2H, s, NH_2_), 7.26 (2H, d, *J* = 8.0 Hz, ArH_A_), 7.42 (2H, d, *J* = 8.0 Hz, ArH_B_), 7.43–7.46 (3H, m, ArH_c_ or H_D_ + H-6), 7.66 (1H, d, *J* = 2.0 Hz, H-8), 7.70 (2H, d, *J* = 8.0 Hz, ArH_c_ or H_D_), 7.80 (1H, s, H-8′), 7.88 (1H, d, *J* = 8.7 Hz, H-6), 8.19 (1H, t, *J* = 6.2 Hz, ArCH_2_*NH*), 8.78 (1H, s, H-4), 8.79 (1H, t, *J* = 5.5 Hz, *NH*CH_2_CH_2_), 10.53 (1H, s, NH), 12.40 (1H, s, NH). δ _C_ (101 MHz, DMSO‑*d*_6_) 24.68 (CH_3_), 38.94 (CH_2_), 41.45 (CH_2_), 66.12 (CH_2_), 66.54 (CH_2_), 68.80 (CH_2_), 69.35 (CH_2_), 70.03 (CH_2_), 70.29 (CH_2_), 83.73 (C), 103.74 (CH), 113.32 (C), 115.22 (CH), 115.46 (C), 127.24 (CH), 127.27 (CH), 128.47 (CH), 131.09 (CH), 134.60 (CH), 135.18 (C), 139.30 (C), 139.42 (C), 144.82 (C), 147.55 (CH), 153.04 (C), 155.16 (C), 159.61 (C), 160.88, (C) 161.33 (C), 169.25 (C). m/z (ESI): Found: 863.3501C_43_H_47_BN_8_O_11_ requires (*M + H)* ^*+*^, 863.3538.

Synthesis of**PeroxyCoum CP**. COMU (53 mg, 0.12 mmol, 1.5 eq) was added to a suspension of 4-[4'-(aminomethyl)benzyloxy]-6-chloropyrimidin-2-amine **12** (33 mg, 0.12 mmol, 1.5 eq), carboxylic acid **10** (50 mg, 0.082 mmol, 1.0 eq) and diisopropylethylamine (43 μL, 0.25 mmol, 3.0 eq) in dry DMF (3 mL). The resulting solution was stirred overnight, concentrated under vacuum then purified by column chromatography using a 12 g Agela cartridge eluting with CH_2_Cl_2_:MeOH (100:0 increasing to 80:20 over 20 column volumes) to give the amide as a cream solid (48 mg, 69 %). *δ*_H_ (400 MHz, CDCl_3_): 1.27 (12H, s, 4 × CH_3_), 3.42–3.53 (4H, m, NH*CH*_*2*_ + OCH_2_), 3.54–3.65 (4H, m, 2 × OCH_2_), 3.99 (2H, s, OCH_2_), 4.47 (2H, d, *J* = 6.0 Hz, NH*CH*_*2*_), 5.07–5.15 (4H, m, OCH_2_ + NH_2_), 5.17 (2H, s, OCH_2_), 6.03 (1H, s, 5′-H), 7.14–7.24 (4H, m, 4 × ArH, H_C_ + H_D_), 7.28 (1H, dd, *J* = 8.5, 2.1 Hz, H-6), 7.32 (2H, d, *J* = 7.6 Hz, 2 × ArH, H_A_ or H_B_), 7.45 (2H, d, *J* = 8.5 Hz, H-5 + NH), 7.53 (1H, d, *J* = 2.1 Hz, H-8), 7.64 (1H, s, NH), 7.75 (2H, d, *J* = 7.6 Hz, 2 × ArH, H_A_ or H_B_), 8.66 (1H, s, H-4), 8.93 (1H, t, *J* = 5.3 Hz, NH).*δ*_C_ (101 MHz, CDCl_3_): 25.00 (CH_3_), 39.48 (CH_2_), 42.61 (CH_2_), 67.65 (CH_2_), 68.11 (CH_2_), 69.63 (CH_2_), 70.17 (CH_2_), 70.70 (CH_2_), 71.11 (CH_2_), 84.10 (C), 97.30 (CH), 105.23 (CH), 114.06 (C), 115.75 (CH), 115.80 (C), 127.62 (CH), 127.91 (CH), 128.32 (CH), 130.70 (CH), 135.09 (C), 135.27 (CH), 138.46 (C), 138.62 (C), 144.13 (C), 148.01 (CH), 152.77 (C), 155.80 (C), 161.03 (C), 161.76 (C), 162.06 (C), 162.31 (C), 170.20 (C), 170.98 (C).Note – C–B carbon not observed. m/z (ESI): Found: 879.2891. C_42_H_46_BClN_6_O_11_Na requires (*M + Na)*^*+*^, 879.2898.

Synthesis of**Coum BG**. PyOxim (101 mg, 0.192 mmol, 2.0 eq) was added to a solution of carboxylic acid **13** (43 mg, 0.096 mmol, 1.0 eq), 6-[4'-(aminomethyl)benzyloxy]-7H-purin-2-amine **11** (31 mg, 0.115 mmol, 1.2 eq), and diisopropylethylamine (85 μL, 0.48 mmol, 5.0 eq) in dry DMF (2 mL). The solution was stirred under argon at R.T. overnight. After this time the solution was concentrated under vacuum then purified by column chromatography using a 12 g Agela cartridge eluting CH_2_Cl_2_:MeOH (100:0 increasing to 75:25 over 10 column volumes) to give the amide as a yellow/green viscous oil (49 mg, 84 %). *δ*_H_ (400 MHz, DMSO‑*d*_6_): 3.44 (2H, apparent q, *J* = 5.5 Hz, NH*CH*_*2*_CH_2_), 3.53 (2H, t, *J* = 5.5 Hz, NHCH_2_*CH*_*2*_), 3.56–3.69 (4H, m, 2 × OCH_2_), 3.95 (2H, s, CH_2_), 4.32 (2H, d, *J* = 6.2 Hz, Ar*CH*_*2*_NH), 5.42 (2H, s Ar*CH*_*2*_O), 6.26 (2H, s, NH_2_), 6.44 (1H, d, *J* = 2.0 Hz, H-8), 6.62 (1H, dd, *J* = 8.6 + 2.1 Hz, H-6), 6.75 (2H, s, NH_2_), 7.27 (2H, d, *J* = 8.1 Hz, ArH_A_), 7.42 (2H, d, *J* = 8.1 Hz, ArH_B_), 7.57 (1H, d, *J* = 8.6 Hz, H-5), 7.81 (1H, s, H-8′), 8.19 (1H, t, *J* = 6.2 Hz, ArCH_2_*NH*), 8.61 (1H, s, H-4), 8.77 (1H, t, *J* = 5.5 Hz, *NH*CH_2_CH_2_), 12.40 (1H, s, NH). *δ*_C_ (101 MHz, DMSO) 38.76 (CH_2_), 41.45 (CH_2_), 66.55 (CH_2_), 68.97 (CH_2_), 69.32 (CH_2_), 70.03 (CH_2_), 70.28 (CH_2_), 97.39 (CH), 108.12 (C), 109.01 (C), 112.60 (CH), 127.26 (CH), 128.47 (CH), 131.83 (CH), 135.17 (C), 139.31 (C), 148.07 (CH), 155.82 (C), 157.22 (C), 159.59 (C), 161.72 (C), 162.19 (C), 169.22 (C). m/z (ESI): Found: 625.2122C_29_H_30_N_8_NaO_7_ requires (*M + Na)* ^*+*^, 625.2130.

Synthesis of**Coum CP**. COMU (80 mg, 0.19 mmol, 1.5 eq) was added to a suspension of -[4'-(aminomethyl)benzyloxy]-6-chloropyrimidin-2-amine **12** (49 mg, 0.19 mmol, 1.5 eq), carboxylic acid **13** (56 mg, 0.12 mmol, 1.0 eq) and diisopropylethylamine (108 μL, 0.62 mmol, 5.0 eq) in dry DMF (3 mL). The resulting solution was stirred overnight, concentrated under vacuum then purified by column chromatography using a 12 g Agela cartridge eluting with CH_2_Cl_2_:MeOH (100:0 increasing to 80:20 over 20 column volumes) to give the amide as a cream solid (53 mg, 70 %). *δ*_H_ (400 MHz, MeOD): 3.47 (2H, q, *J* = 5.2 Hz, CH_2_), 3.59 (2H, t, *J* = 5.2 Hz, CH_2_), 3.63–3.76 (4H, m, 2 × CH_2_), 4.06 (2H, s, CH_2_), 4.45 (2H, s, CH_2_), 5.22 (2H, s, CH_2_), 6.06 (1H, s, H-5′), 6.45 (1H, d, *J* = 2.1 Hz, H-8), 6.65 (1H, dd, *J* = 8.6, 2.1 Hz, H-6), 7.26 (4H, br s, H_A_ + H_B_), 7.40 (1H, d, *J* = 8.6 Hz, H-5), 8.53 (1H, d, *J* = 0.7 Hz, H-4). *δ*_C_ (101 MHz, MeOD): 40.32 (CH_2_), 43.24 (CH_2_), 68.89 (CH_2_), 70.39 (CH_2_), 71.12 (CH_2_), 71.26 (CH_2_), 72.09 (CH_2_), 96.64 (CH), 99.13 (CH), 110.12 (C), 110.16 (C), 114.20 (CH), 128.50 (CH), 129.33 (CH), 132.83 (CH), 136.67 (C), 139.67 (C), 149.64 (CH), 157.69 (C), 159.15 (C), 161.72 (C), 163.96 (C), 164.42 (C), 165.22 (C), 172.32 (C), 172.78 (C). m/z (ESI): Found: 619.1673. C_28_H_29_ClN_6_O_7_Na requires (*M + Na)*^*+*^, 619.1678.

### 2.2 Cloning procedures

Plasmids containing the SNAP tag (pSNAP_f_ #N9183S) and HaloTag (pHTC HaloTag® CMV-neo #G7711) for mammalian expression were purchased from NEB and Promega respectively. Human mitochondrial protein sequences for tagging with SNAP and HaloTag were amplified by PCR from reverse transcribed human RNA purified from HEK293 cells using reverse transcription kit (Omniscript RT, Qiagen) and TriZol RNA purification kit (Invitrogen). The PCR products were directly cloned into the pCR2.1 TOPO-TA cloning vector (Invitrogen #450641). Once the correctness of the sequences was confirmed by Sanger sequencing, the pCR2.1 vectors containing the inserts were digested with *Nhe*I and *Eco*RI prior to ligation into the multiple cloning site of either the pSNAP_f_ or the pHTC vector pre-digested with the same enzymes.

### 2.3 Cell culture

All cells were cultured in Dulbecco's modified Eagle's medium (DMEM, Gibco #11965092) supplemented with 4 mM l-glutamine, 1 mM sodium pyruvate, 10 % v/v fetal bovine serum (FBS) and either 4.5 g/L glucose or 2 g/L galactose. For microscopy, phenol red containing media was replaced with phenol red-free DMEM (Gibco #21063029) supplemented as before. Cells were grown at 37 °C in a controlled atmosphere containing 5 % CO_2_.

### 2.4 Transfection

Wild type HeLa cells were transfected using Fugene HD transfection reagent (Promega #E2311). Cells were seeded at ∼30 % confluency and allowed to expand until 80 % confluent. At this point, old media was replaced with 2 mL fresh DMEM and plates placed back in the incubator. Meanwhile, plasmid DNA was added to a fresh 1.5 mL Eppendorf tube at a final quantity of 4 μg. OptiMEM transfection media, prewarmed to room temperature, was then added to the DNA so that the final volume after addition of all components would be 200 μL. Room temperature Fugene transfection reagent was then added at a 3:1 ratio to DNA (12 μL). Care was taken to avoid unnecessary contact of Fugene reagent with the walls of the Eppendorf tube prior to mixing. The resulting solution was incubated at room temperature for 15 min. Following incubation, 200 μL of transfection solution was added to every required well of the previously prepared 6-well plate, mixed gently and returned to the incubator.

After 24 h, media was replaced with fresh DMEM supplemented with 0.4 mg/mL geneticin for the selection of cells transfected with pSNAP_f_ plasmids. Media was then replaced every 48 h until cell density reached ∼80 % confluency. Cells were then transferred to a fresh T75 flask by trypsinization and left to expand before being frozen in DMEM supplemented with 10 % v/v DMSO for storage or used for experiments directly.

For transient experiments cells were grown in 2-well chamber slides and volumes of transfection reagents were half the amount listed above. Cells were left for 48 h and then visualized using confocal microscopy.

### 2.5 Sub-fractionation by mitoplasting

Stably expressing cell lines were grown in 10 × 10 cm^2^ dishes until ∼80 % confluency was reached. Cells were washed with PBS before being scraped and collected in 5 mL PBS. Cells were spun at 800×*g* for 5 min. Pellets were resuspended in 3 mL of solution A (20 mM HEPES-KOH pH 7.6, 220 mM Mannitol, 70 mM Sucrose, 1 mM EDTA and 0.5 mM freshly prepared PMSF) and incubated on ice for 15 min. Cells were then homogenized using a Teflon homogenizer for 30 strokes on ice and spun at 800×*g* for 5 min at 4 °C to remove cell debris. Supernatant was then spun at 10,000×*g* for 10 min at 4 °C to separate mitochondria (supernatant containing the cytosol was saved). The mitochondrial pellet was resuspended in equal volume of solution A and centrifuged again at 800×*g* for 5 min to remove any residual cell debris. The supernatant was then centrifuged at 10,000×*g* for 20 min at 4 °C before resuspending the mitochondrial pellet in 200 μL of solution A. Mitochondria were separated into 4 fresh Eppendorf tubes equally and centrifuged at 10,000×*g* for 10 min at 4 °C. Pellets were resuspended in either isotonic (250 mM sucrose, 1 mM EDTA, 10 mM Tris-HCl pH 7.6) or hypotonic (1 mM EDTA, 10 mM Tris-HCl pH 7.6) buffers with and without 50 μg/mL proteinase K and incubated on ice for 30 min. PMSF was then added to a final concentration of 2 mM and incubated for a further 10 min on ice. Samples were spun at 12,000×*g* for 5 min at 4 °C and supernatants were collected in fresh tubes. Pellets were resuspended in 10 μL of 2x SDS sample buffer containing 2 mM PMSF and were boiled at 95 °C for 3 min. Supernatants were treated with 10 % (v/v) TCA and incubated on ice for 20 min to precipitate proteins. Precipitated protein was collected by centrifugation at 16,000×*g* for 20 min at 4 °C and pellets were resuspended as before.

### 2.6 Immunofluorescence

Cells were seeded at ∼30 % confluency in 2-well chamber slides coated with poly-l-Lysine. Following 24 h of incubation cells were around 50 % confluent and were prepared for immunofluorescence staining. Cells were treated with 500 nM mitotracker deep red (Invitrogen #M22426) in fresh DMEM for 30 min followed by replacement with label-free DMEM for a further 30 min. Cells were then fixed in 4 % v/v paraformaldehyde for 15 min at room temperature, washed 3 times with PBS for 5 min and solubilised for 10 min with 0.2 % v/v Triton X-100 in PBS. Following initial solubilisation, cells were blocked for 1 h in solution containing 2 % w/v bovine serum albumin (BSA) and 0.2 % v/v Triton X-100 dissolved in PBS. After blocking, cells were incubated overnight at 4 °C with 1:500 dilution of SNAP primary antibody (NEB #P9310S) in 2 % w/v BSA in PBS. The next day cells were washed 3x in PBS at room temperature for 15 min and incubated with 1:500 goat anti-rabbit IgG secondary antibody conjugated to Alexa Fluor 488 (Invitrogen #A32731) in PBS for 1 h. Cells were washed 3 times for 5 min in PBS to remove unbound secondary antibody and treated for 15 min with Nuc Blue DNA stain (Invitrogen #R37605) to counterstain nuclei. Following this, cells were imaged directly using a confocal microscope (details described below).

### 2.7 *In cellulo* PeroxyCoum and coum conjugation to SNAP-tag and treatment with H_2_O_2_ or antimycin A

For live cell measurements of fluorescence from PeroxyCoum CP and Coum CP/BG treated cells expressing SNAP-tag, fluorescence cells were seeded in 24-well plates (Ibidi #82426) at 30,000 cells per well. After 24 h, cells were treated for 20 min with 100 nM TMRE in DMEM to stain functional mitochondria. DMEM was replaced for 30 min before cells were treated with 5 μM PeroxyCoum CP or Coum CP/BG in DMEM for 30 min. Unbound probe was removed by washing once with phenol red-free DMEM before measurement by confocal microscopy. Treatments were performed by mixing a solution containing 2x the desired dose of either H_2_O_2_ or antimycin A in an equal volume in each well to give a final 1x concentration.

### 2.8 Microscopy

All microscopy was performed using a Zeiss LSM 880 confocal microscope and image analysis was performed using FIJI v.2.1.0/1.53f51. For immunofluorescence, slides were analyzed using a 63× oil immersion objective. Hoechst stain was excited with a 405 nm diode, AF488 with a 488 nm Argon laser and Mitotracker deep red with a 633 nm HeNe laser. Images were analyzed and merged using FIJI.

For live cell imaging, coumarin was excited using the 405 nm diode while TMRE was excited using 561 nm DPSS and images collected using a 10× objective to capture many cells. Positional data were stored for each of the 24-wells containing cells so that the same cells were imaged across all timeseries. A zero timepoint was measured for each well prior to addition of any treatments which would act as the F_0_ for normalization purposes. Each experiment was performed at least three times and quantification is a mean value for >30 cells from each experiment plotted as a boxplot.

### 2.9 Data and statistical analysis

All microscopy data were analyzed using FIJI with quantitative coumarin fluorescence data being measured by running an in-house script for individual cell detection and of pixel intensity. All statistical analyses were performed in R version 4.0.2 with significance values as stated in figure legends.

## 3 Results

### 3.1 Chemical synthesis and initial characterization of PeroxyCoum dyes

**Fig. 2:**
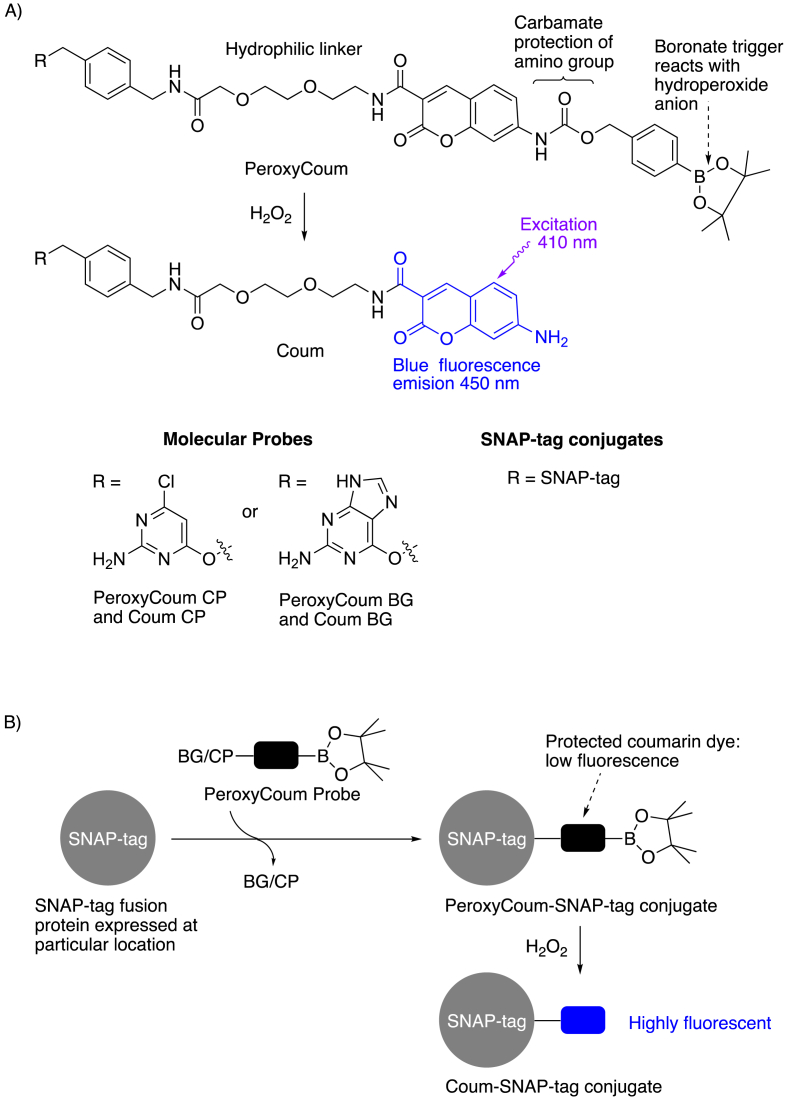
The molecular probes and SNAP-tag conjugates used in this study (A) The PeroxyCoum derivatives react with the hydroperoxide anion from hydrogen peroxide to generate the highly fluorescent Coum derivatives. (B) A PeroxyCoum probe generates a PeroxyCoum-SNAP-tag conjugate from SNAP-tag expressed at a particular intracellular site. The conjugate then reports on hydrogen peroxide at that site.

The aim of the work was to position a non-fluorescent sensor at specific sites in the mitochondria. This would then react with hydrogen peroxide to produce a fluorescent reporter at that site. We chose amides of the 7-amino-coumarin 3-carboxylic acid fluorophore (Coum, Fig. 2A) as the reporter. This bright fluorophore fluoresces blue when it is excited with visible violet light. The sensor would use a well-validated switch on mechanism whereby the amino group is protected as a benzylcarbamate bearing a H_2_O_2_-sensitive boronate trigger [29] (PeroxyCoum, Fig. 2A). The absorption and emission spectra of the PeroxyCoum carbamate is blue-shifted relative to the Coum dye. As such, PeroxyCoum has negligible blue fluorescence when excited with violet light. Upon reaction with hydrogen peroxide PeroxyCoum fragments to release the highly fluorescent Coum reporter. Initially SNAP-tag was chosen as the self-labeling protein and so the complementary tag was incorporated into the molecular probe that would react with it to attach the PeroxyCoum sensor. The complementary tag can be benzylguanine (BG) or benzylchloropyrimidine (CP). Both PeroxyCoum BG and PeroxyCoum CP molecular probes were synthesized bearing a BG tag or CP tag attached to the coumarin 3-carboxy group via a short linker (Fig. 1 for synthesis). The corresponding free amine fluorescent dyes Coum BG and Coum CP were also synthesized (Fig. 1). These would be used in experiments to determine the localization of SNAP-tag and to reveal the maximum response possible from the corresponding PeroxyCoum-SNAP-tag conjugates.

The overall concept of the sensing system is shown in Fig. 2B. The SNAP-tag would be expressed as a fusion protein that would localize to a particular site in each cell line. The SNAP-tag expressing cells would then be treated with the PeroxyCoum molecular probe (BG or CP) to form PeroxyCoum-SNAP-tag conjugates at the site of interest. The cells would be washed to remove any excess molecular probe. The sensor would then be ready to respond to hydrogen peroxide under the conditions of each experiment. The blue fluorescence increase in response to hydrogen peroxide would be integrated over time to report on the hydrogen peroxide at that site under the particular experimental conditions.

**Fig. 3:**
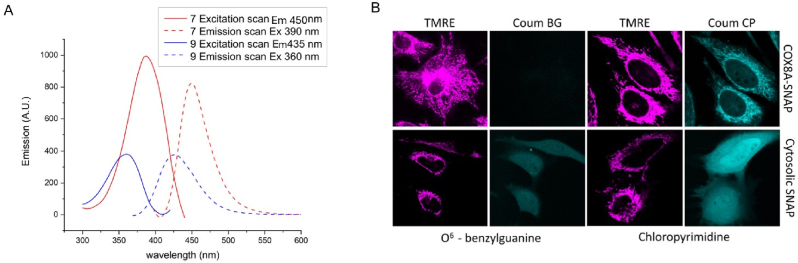
Validation of coumarin derivatives (A) Excitation and emission spectra of a free aminocoumarin dye **7** and the corresponding carbamate **9** (see Fig. 1 for structures). 1 μM in PBS pH 7.4. (B) Representative confocal microscopy images of Coum BG and Coum CP binding to cytosolic and mitochondrial matrix targeted SNAP-tag. The membrane potential dependent probe, TMRE, is used as a mitochondria label.

First we validated our probes. For simplicity the fluorescent properties of a 7-amino-coumarin 3-carboxamide and its protected carbamate derivative were determined using synthetic precursors (compounds **7** and **9**, Fig. 1, respectively). As expected the carbamate **9** absorbs and emits at shorter wavelengths than the free amine **7** (Fig. 3A). As a result the aminocoumarin **7** emits about 20 times more brightly at 450 nm than the carbamate when each is excited at 410 nm (Fig. S2), confirming that the switch on reporting would be effective. Successful reaction of the carbamate **9** with H_2_O_2_ to give the free aminocoumarin was then demonstrated by monitoring the increase in absorption at 405 nm under pseudo first order conditions (Fig. S3). Next the cell permeability and intracellular conjugation of the chloropyrimidine and benzylguanine derivatives to SNAP-tag was investigated using Coum CP and Coum BG. Cells expressing SNAP-tag in the mitochondria and cells expressing SNAP-tag in the cytosol were treated with these molecular probes (Fig. 3B, mitochondria labelled with TMRE). Coum CP gave substantially better fluorescent labelling so only CP derivatives were used in later experiments. The scale provided is semi-quantitative, and the images show a very clear difference in pixel intensity between the Coum BG and Coum CP samples. The Conjugation of Coum to purified SNAP-tag protein was also demonstrated with Coum CP (Fig. S4). Our results are consistent with literature precedent [22]: both the BG and CP tags can be used to conjugate dyes to SNAP-tag but the chloropyrimidine derivatives have been shown to cross the plasma membrane more easily [22].

### 3.2 SNAP-tagged mitochondrial proteins and precursors allow sorting of the tag to defined subcellular and sub-mitochondrial compartments

**Fig. 4:**
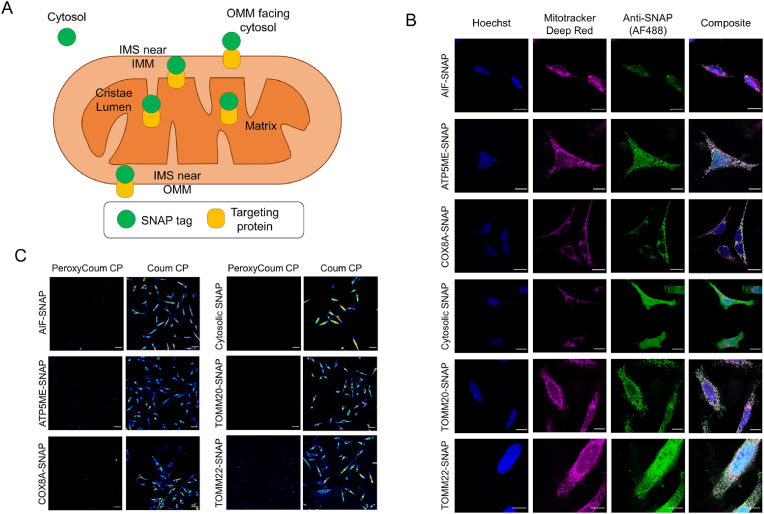
SNAP tags allow the targeting of chloropyrimidine coumarin to distinct mitochondrial sub-compartments in HeLa cells (A) Schematic indicating the expected subcellular and sub-mitochondrial localizations of SNAP-tags. (B) Representative immunofluorescence confocal microscopy images of fixed HeLa cells stably expressing SNAP-tags. Cells were treated with Hoechst for nuclear staining (blue), Mitotracker deep red for mitochondrial staining (magenta) and labelled with anti-SNAP primary antibody and an AF-488 labelled secondary antibody (green). Composite images indicate co-localization with mitochondria for all cell lines except cytosolic SNAP, as expected. Scale bars are 20 μm (C) Representative confocal microscopy images of SNAP-tag expressing HeLa cells labelled with either caged (non-reacted) PeroxyCoum CP or free (amine product) Coum CP to determine binding and fluorescence enhancement that would be expected in the presence of H_2_O_2_. Scale bars are 50 μm.

In order to obtain spatially accurate data on the distribution and transport of ROS (specifically H_2_O_2_) within mitochondria, we first needed to correctly localize the coumarin-based fluorescent reporter to the various sub-compartments of the organelle using the SNAP-tags. Six chimeric constructs were designed coding for the SNAP-tag (182 amino acid residues) fused C-terminally to the N-terminal targeting sequences of: (1) TOMM20 for localization at the outer mitochondrial membrane facing the cytosol; (2) TOMM22 for attachment to the outer membrane facing the IMS; (3) AIFM1 (AIF) for attachment to the IM facing the IMS; (4) ATP5ME for localization within the IM cristae lumen; and (5) COX8A for localization of the SNAP-tag in the matrix. Finally, a sixth construct containing only the SNAP-tag coding sequence, was also generated to allow synthesis of the soluble SNAP-tag and retention within the cytosol with no mitochondrial targeting (Fig. 4A).

These constructs were transfected into wild type HeLa cells and stable cell lines were generated using a geneticin selection marker present in the plasmid. The mitochondrial function of all stable cell lines was the same as un-transfected control cells as measured by Seahorse oxygen consumption rate assays (Fig. S5). These were done in triplicates for statistical validation and normalization was done with respect to cell density [30] quantified by NucBlue (Invitrogen) nuclear stain fluorescence using a BMG Labtech POLARstar OMEGA plate reader. The correct sub-mitochondrial localization of the different SNAP-tags was confirmed using both immunofluorescence imaging of fixed cells and biochemical localization using sub-fractionation and proteinase K treatment of purified mitochondria and mitoplasts (Fig. 4B and Fig. S6). For immunofluorescence imaging, the mitochondria in the cells were stained with 500 nM Mitotracker Deep Red prior to fixation, followed by permeabilization and antibody binding. Colocalization of fluorescent signals from the SNAP-tag antibody and the Mitotracker dye indicated that all constructs, except for the cytosolic SNAP-tag, were localized correctly to mitochondria (Fig. 4B and Fig. S6).

To further confirm the precise submitochondrial localization of the SNAP-tags, mitochondria from the stable HeLa cell lines were made, and purified and further sub-fractionated into mitoplasts using hypo-osmotic buffer conditions in the presence and absence of proteinase K. The resulting fractions, as well as the intact mitochondrial fractions, were analyzed by SDS-PAGE and western blotting and compared with specific sub-compartment markers. All constructs showed a banding pattern indicative of correct cellular and sub-mitochondrial localization (Fig. S6).

### 3.3 Binding of caged (PeroxyCoum CP) and free (coum CP) chloropyrimidine coumarin to SNAP expressing cells

To determine the feasibility of using the conversion of PeroxyCoum to Coum to assess mitochondrial ROS dynamics, we first bound the unreacted (PeroxyCoum CP) and free-amine (Coum CP) probes to each of our cytosolic and sub-mitochondrial SNAP-tag expressing cell lines. Cells treated with Coum CP probes showed high levels of fluorescence for each of the cell lines, for which the correct sub-mitochondrial localization of the SNAP-tag was assessed by biochemical mitochondria sub-fractionation assays (Fig. S6). This was in contrast to low signals detected from cells treated with the unreacted PeroxyCoum probes, as expected, given the lack of oxidative stress being imposed on the cells (Fig. 4C). Coum CP treated cells showed around a 10-fold higher fluorescence compared to those treated with the unreacted PeroxyCoum CP probe in all sub-mitochondrial locations except for the outer membrane facing the IMS (TOMM22) and the cristae lumen (ATP5ME), where a 5-fold higher fluorescence was measured (Fig. S7). Conjugation with PeroxyCoum was demonstrated by competition experiments in which pre-treatment with PeroxyCoum CP prevented localization of Coum CP (Fig. S8).

Together, these data indicated that CP coumarins are able to bind to both cytosolic and mitochondrially localized SNAP-tagged proteins. Importantly, it further showed that the fluorescence difference between PeroxyCoum CP and Coum CP when excited at 405 nm would be large enough to allow measurement of H_2_O_2_ flux within cells and mitochondria exposed to oxidative stress conditions.

### 3.4 PeroxyCoum CP can detect ROS with sub-mitochondrial resolution upon long and short term exposure of cells to H_2_O_2_

**Fig. 5:**
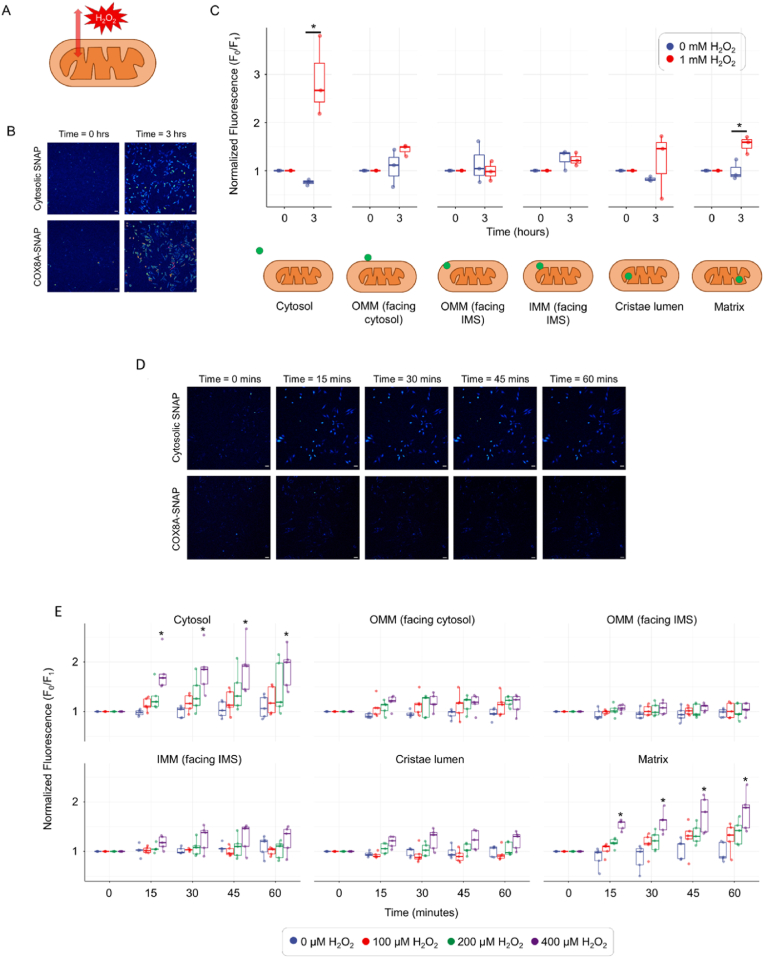
PeroxyCoum CP can detect exogenous changes in H_2_O_2,_ in both high and low exposure levels, within the different mitochondrial sub-compartments (A) Schematic representation showing the exogenous supply of H_2_O_2_· (B) Representative confocal microscopy images of cytosolic and matrix targeted SNAP-tag expressing HeLa cells treated with 1 mM of exogenous H_2_O_2_ for 3 h. Cyan color represents coumarin fluorescence detected using 405 nm excitation. False color representation is the same as in Fig. 3. Scale bars are 50 μm (C) Normalized fluorescence intensity arising from H_2_O_2_ oxidation of PeroxyCoum conjugates in various mitochondrial sub-compartments. Data points represent mean values from >30 individual cells taken across three independent experiments, normalized to fluorescence at time = 0. Asterisks represent statistical significance (p < 0.05) using two-way ANOVA with Tukey (HSD) posthoc analysis. (D) Representative confocal microscopy images of cytosolic and matrix targeted SNAP-tag expressing HeLa cells treated with 400 μM of exogenous H_2_O_2_ for up to 60 min with images captured every 15 min. Cyan color represents coumarin fluorescence detected using 405 nm excitation. Scale bars are 50 μm (E) Normalized fluorescence intensity of Coum conjugates generated from PeroxyCoum conjugates in various mitochondrial sub-compartments. Data points represent mean values from >30 individual cells taken across five independent experiments, normalized to fluorescence at time = 0. Asterisks represent statistical significance (p < 0.05) using two-way ANOVA with Tukey (HSD) posthoc analysis.

Initially, we aimed to assess the effects of high concentrations of ROS-generating compounds on the fluorescence arising from the generation of the Coum-SNAP-tag conjugate from the PeroxyCoum-SNAP-tag conjugate at the various subcellular locations previously discussed. We therefore treated cells containing PeroxyCoum-SNAP-tag conjugates for 3 h with 1 mM of exogenously applied H_2_O_2_^31^ (Fig. 5A).

Treatment of cells with 1 mM H_2_O_2_ for 3 h led to significantly increased fluorescence in both the cytosol and the mitochondrial matrix, indicating that the PeroxyCoum was indeed sensitive to high levels of H_2_O_2_ stress and generated the fluorescent Coum dye when bound to SNAP-tags in different cellular compartments. Fluorescence signals, normalized to those collected at time zero, were increased almost 4 times in the cytosol (from 0.76 ± 0.03 to 2.89 ± 0.39), while matrix localized fluorescence was increased approximately 1.5 times (from 0.99 ± 0.10 to 1.55 ± 0.09) following 3 h of treatment (Fig. 5B and C). Interestingly, no significant increase in the fluorescence intensity from reaction of PeroxyCoum conjugates was observed at the OMM surface or IMS locations (either the bulk IMS from the OMM side, or the IMM side or indeed the lumen of the cristae). A similar low response in the IMS has been reported using the fluorescent protein H_2_O_2_ sensor HyPer7 [15].

Next, we wanted to assess the utility of PeroxyCoum CP for the detection of lower levels of H_2_O_2_ across shorter timescales. We therefore treated cells with different concentrations of exogenous H_2_O_2_, followed by measurement of fluorescence emission from the Coum conjugates generated by H_2_O_2_ oxidation of the PeroxyCoum conjugates across minute rather than hour timescales. We were limited in measuring shorter temporal kinetics due to image acquisition times using our confocal microscope with the 24-well plate experimental design that we used for high throughput.

As observed previously, treatment of cells with exogenous H_2_O_2_ led to increased fluorescence from H_2_O_2_ oxidation of PeroxyCoum conjugates in the cytosol and matrix but showed little change when localized in the IMS or at the OMM (either on the cytosolic side or the IMS side) (Fig. 5D and E). Significant increases in fluorescence (p < 0.05) were observed when using 400 μM of H_2_O_2_ at all the tested time points, whereas with lower concentrations there was an increasing trend but not reaching the threshold for statistical significance (Fig. 5D and E). We noted that the boronate probes react with HOO^−^ rather than with H_2_O_2_ itself (pKa = 11.6) [31] and that the IMS has a lower pH than the cytosol and matrix. Therefore, we investigated the intrinsic effect of pH on the reaction between PeroxyCoum derivatives and H_2_O_2_ (Supplementary Figs. S9 and S10). We found that the dye itself is not sensitive to pH (Fig. S9), but the reaction to release it is very pH-sensitive, with reaction at pH 8.0 approximately 12 times faster than reaction at pH 6.5 (Fig. S10).

### 3.5 PeroxyCoum CP can detect ROS with sub-mitochondrial resolution using endogenous mitochondrial ROS production by treatment with antimycin A

**Fig. 6:**
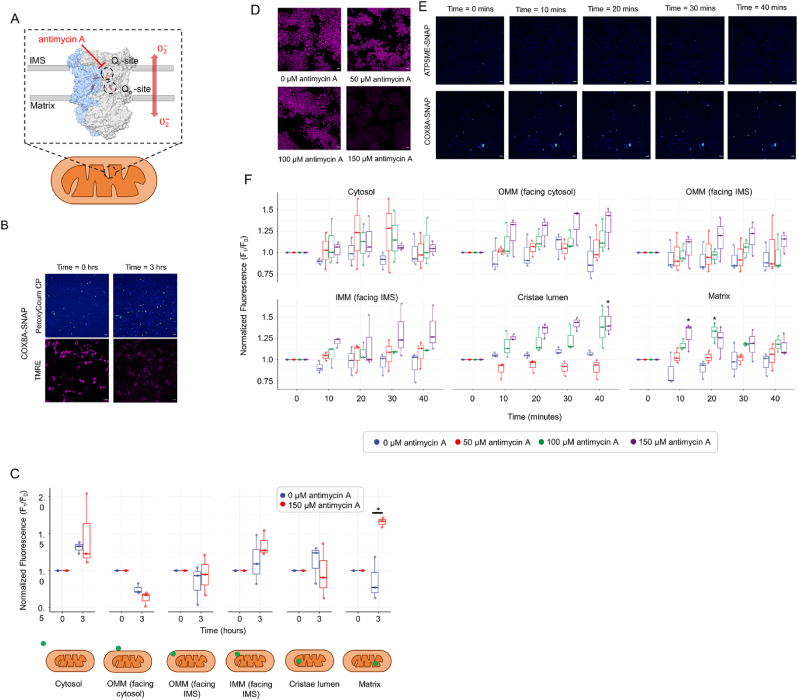
Exposure to antimycin A reveals distinct coumarin fluorescence patterns when in long-term exposure (A–C) compared to short-term - exposure (D–E) (A) Schematic representation of O_2_^•-^ production sites within complex III upon antimycin A treatment. Antimycin A blocks the Q_i_ site resulting in the accumulation of semiquinone at site Q_o_ resulting in O_2_^•-^ release to both the matrix and the IMS [9]. (B)Representative confocal images as in A of matrix targeted SNAP-tag HeLa cells treated with 150 μM antimycin A for 3 h. False color representation is the same as in previous Figures. Scale bars are 50 μm (C) Quantification of coumarin fluorescence as in Fig. 5C. Asterisks represent statistical significance (p < 0.05) using two-way ANOVA with Tukey (HSD) posthoc analysis. (D) Representative confocal microscopy image of the effects of 150 μM antimycin A treatment on the fluorescence of TMRE. Scale bars are 50 μm (E) Representative confocal microscopy images of PeroxyCoum CP treated HeLa cells expressing SNAP-tag in the cristae lumen and matrix when these are treated with 150 μM of exogenous H_2_O_2_ for up to 40 min with images captured every 15 min. Cyan color represents coumarin fluorescence detected using 405 nm excitation. Scale bars are 50 μm (F) Normalized fluorescence intensity from generation of Coum conjugates from PeroxyCoum conjugates in various mitochondrial sub-compartments. Data points represent mean values from >30 individual cells taken across three independent experiments, normalized to fluorescence at time = 0. Asterisks represent statistical significance (p < 0.05) using two-way ANOVA with Tukey (HSD) posthoc analysis.

Given that the majority of mitochondrial ROS is generated from within the organelle and the first ROS formed is O_2_^•-^ rather than H_2_O_2_, we also wanted to test the fluorescence response of PeroxyCoum-SNAP-tag conjugates when ROS production was stimulated from inside the mitochondria. Antimycin A is a complex III inhibitor which results in the release of superoxide O_2_^•-^ free radicals both to the matrix and the IMS [9,32] (Fig. 6A). O_2_^•-^ is quickly dismutated to H_2_O_2_ by the superoxide dismutase enzymes SOD1 and SOD2, in the IMS and matrix respectively. Cells were thus treated with 150 μM of antimycin A for 3 h and the fluorescence of the Coum conjugates generated by H_2_O_2_ oxidation of the PeroxyCoum conjugates was measured as before. The fluorescence of TMRE, a mitochondrial membrane potential dye used in all experiments to track cell and mitochondrial position, was significantly reduced following Antimycin A treatment, indicating the efficacy of the treatment for inducing loss of the inner membrane electrochemical potential, which in this case is concomitant with high levels of ROS production (Fig. 6B). Thus, a significant, approximately two-fold, increase of fluorescence (from 0.86 ± 0.14 to 1.66 ± 0.03) (presumably linked to H_2_O_2_ increase) was detected in the matrix when SNAP was localized in the matrix using the COX8A mitochondrial targeting sequence (Fig. 6C). Again, no significant increase in the fluorescent signals were detected in the IMS or the cristae lumen and, under these conditions, no release of H_2_O_2_ to the cytosol was detected, compared with the control cells incubated without Antimycin A (Fig. 6C).

However, in contrast to treatment with exogenous H_2_O_2_, short timescale treatment with varying concentrations of antimycin A showed significantly different trends in dye fluorescence as opposed to long term treatment. TMRE fluorescence was again affected by antimycin A treatment albeit only with the concentration of 150 μM providing a clear signal change (Fig. 6D). Significant increases in Coum fluorescence were this time detected in the cristae lumen as well as in the matrix. Fluorescence in the matrix increased within 10 or 20 min following antimycin A treatment, before leveling off. Coum fluorescence in the cristae lumen showed an increasing trend over time until the end of the experiment at 40 min (Fig. 6E and F). Given that we saw limited dye fluorescence in the cristae lumen upon long term exposure, this would suggest a quick, detectable response takes place in this sub-compartment following treatment, but then no further reaction of the PeroxyCoum conjugate occurs. This might be due to cristae remodeling or removal of H_2_O_2_ by an influx of peroxidase enzymes from the bulk IMS, or indeed a combination of the two. Background fluorescence from the control cells also increases over longer timescales possibly masking fluorescence changes at these timepoints (Fig. 6F). It is interesting that no significant release of H_2_O_2_ from inside the mitochondria was detected either at the level of the OMM or the cytosol, albeit there was an increasing trend in dye fluorescence at the level of both the IMS and the outer leaflet of the OMM suggesting that low levels of H_2_O_2_ are indeed released (Fig. 6F).

### 3.6 Cell growth in increased respiratory conditions displays altered H_2_O_2_ flux

Many previous studies have shown variability in H_2_O_2_ dynamics between cell lines and under different metabolic conditions [15,33]. We therefore assessed the response of PeroxyCoum treated SNAP-tag expressing HeLa cells grown using galactose as the primary carbon source. Under these growth conditions glycolysis is limited whilst mitochondrial respiratory function is enhanced to provide the necessary energy to cells [34]. Thus, cells were cultured in media containing 2 g/L galactose for 3 days prior to treatment with exogenous H_2_O_2_ and Antimycin A.

**Fig. 7:**
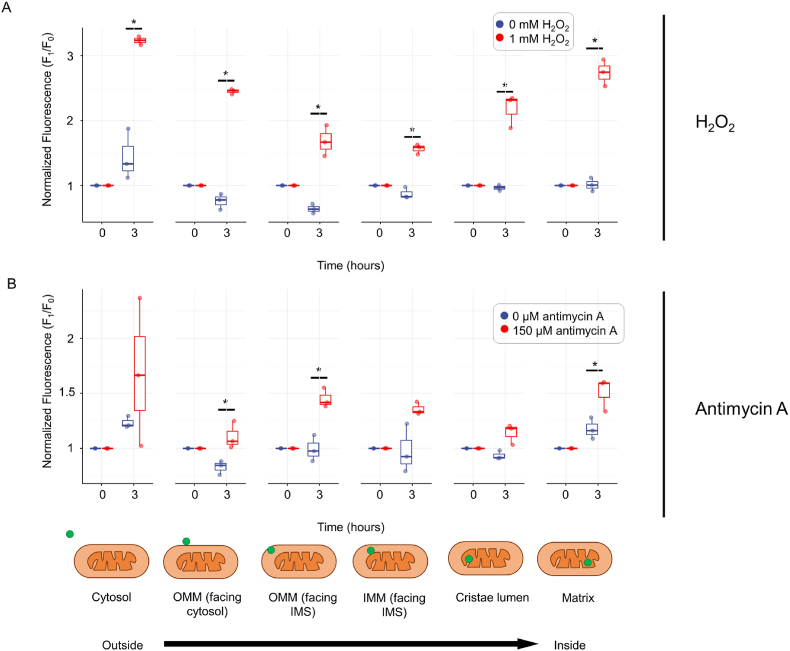
Cells grown in galactose display altered coumarin responses to H_2_O_2_ and antimycin A (A) Normalized fluorescence intensity of H_2_O_2_ oxidized PeroxyCoum conjugates in various mitochondrial sub-compartments. Data points represent mean values from >30 individual cells taken across five independent experiments, normalized to fluorescence at time = 0. Asterisks represent statistical significance (p < 0.05) using one-way ANOVA with Tukey (HSD) posthoc analysis. (B) Normalized fluorescence intensity of H_2_O_2_ oxidized PeroxyCoum conjugates in various mitochondrial sub-compartments. Data points represent mean values from >30 individual cells taken across five independent experiments, normalized to fluorescence at time = 0. Asterisks represent statistical significance (p < 0.05) using one-way ANOVA with Tukey (HSD) posthoc analysis.

Cells grown in galactose medium showed a greater susceptibility to oxidative changes than the cells grown in glucose medium when treated with the same concentrations of exogenous H_2_O_2_ and Antimycin A. Quantification of fluorescence from H_2_O_2_ oxidation of PeroxyCoum conjugates showed significant increases in intensity across all subcellular and sub-mitochondrial compartments following treatment with exogenous H_2_O_2_ (Fig. 7A). This was a marked change from the detection pattern determined in cells grown in glucose medium, and it suggests that higher rates of respiration and possibly changes in reductive pathways are responsible, especially within the IMS and in close proximity to the OMM.

When internal mitochondrial ROS production was stimulated using Antimycin A, we were able to detect significant increases in H_2_O_2_ levels in the matrix and IMS, as well as in the cytosol within close proximity to the OMM (Fig. 7B) indicating H_2_O_2_ release to the cytosol under these conditions. There were also non-significant increases in fluorescence in the cristae lumen and the cytosol suggesting that H_2_O_2_ is likely released into all of these compartments upon antimycin A treatment but at levels below the detection limit of the current probe (Fig. 7B). It should be noted that there was a high degree of heterogeneity between experiments for cytosolic samples and this may have been due to poor TMRE uptake by these cells. TMRE was used as a marker for subsequent image analysis and as such low levels of TMRE meant fewer cells could be analyzed.

### 3.7 Targeting of multiple probes to distinct subcellular and sub-mitochondrial locations

**Fig. 8:**
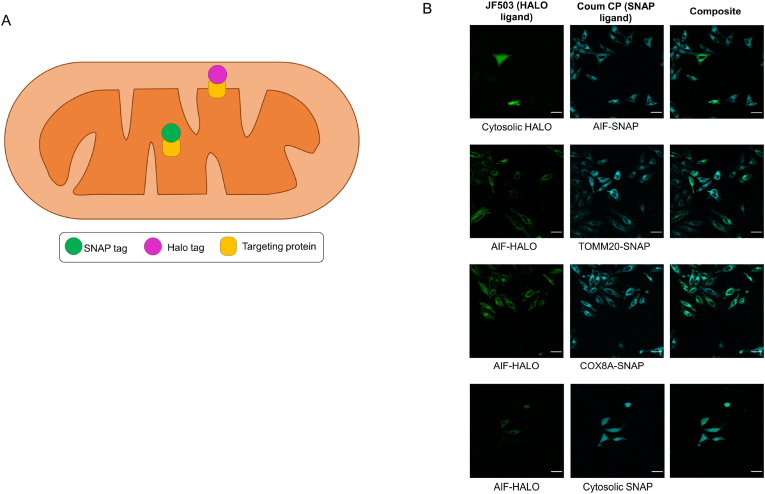
Concomitant use of SNAP-tag and HaloTag allows the dual labelling of different sub-compartments with spectrally distinct dyes (A) Schematic representation of dual labelling strategy (B) Representative confocal microscopy images showing binding of a green fluorescent Janelia-Fluor 503 (JF503) bearing the chloroalkane tag recognized as a ligand by HaloTag and Coum CP in stable SNAP-tag cell lines transiently transfected with the relevant HaloTag containing constructs. Scale bars are 50 μm.

Targeting multiple probes to separate sub-mitochondrial locations within the same cell would be very valuable to provide a read out on the dynamics of multiple ROS molecules simultaneously in the same cell. To demonstrate that double labelling is possible (as a proof of principle experiment), we transiently expressed fusion proteins containing the different mitochondrial targeting sequences on HaloTag, into our stably expressing SNAP-tag cell lines for dual labelling. The HaloTag is derived from a bacterial haloalkane dehalogenase enzyme and can form covalent bonds with small molecules containing the complementary chloroalkane tag [21]. Since HaloTag recognizes a chemically distinct tag in the small molecule, this approach allows for the independent, but concurrent, labelling of both SNAP-tag and HaloTag with spectrally distinct fluorophores using two fluorescent probes each specifically tagged for recognition by only one of the self-labelling proteins. In this way we were able to target two dyes to separate sub-compartments of the mitochondria in the same cells (Fig. 8). The differential labelling demonstrates that it may be possible to target two distinct sensors that report at different fluorescent wavelengths to allow simultaneous measurements of multiple distinct chemical species within different subcellular and sub-mitochondrial locations within the same cell, experiencing the same treatment conditions. These interesting further applications are considered in the Discussion part.

It should be noted that the HaloTag was less easy to work with than the SNAP-tag in our hands. Attempts to generate HeLa cell lines stably expressing HaloTag proteins were not successful. Also, transient transfection and mitochondrial targeting was heavily dependent on the specific mitochondrial targeting signal that was used. For example, the well characterized matrix targeting sequence of COX8A was unable to deliver the HaloTag fusion protein to mitochondria (in contrast to very efficiently delivering the SNAP-tag), while the IMS localized AIF-Halo showed clear mitochondrial localization (Fig. 8).

## 4 Discussion

The dynamics of H_2_O_2_ flux through mitochondrial sub-compartments, as well as its subsequent release to the cytosol, is a key physiological process that allows cells to realize the beneficial and protective signalling effects of H_2_O_2_ [[35], [36], [37]]. Here we teased out specific H_2_O_2_responses to different metabolic states dictated by different nutrients, with precise, sub-mitochondrial resolution. We synthesized and characterized a new set of small molecule probes that allowed us to determine precise dynamic changes in H_2_O_2_ in response to metabolic alterations induced by the different nutrients, in specific sub-mitochondrial compartments. We find that cells grown in galactose-based media (leading to increased respiratory conditions) compared to those grown in glucose (i.e., in more glycolytic conditions) are characterized by increased H_2_O_2_ flux in conditions favoring respiration. In this work we focused on H_2_O_2_ as one critical species for mitochondrial metabolism and signaling, but the approach can be expanded to many other species of interest for mitochondrial metabolism.

Previous approaches utilizing small chemical probes to monitor H_2_O_2_ flux have been informative but are limited in their study of this phenomenon due to the lack of specific targeting to defined mitochondrial sub-compartments, other than the matrix. Recent studies using the highly sensitive fluorescent protein reporter HyPer7 have overcome some of these issues through specific targeting of this protein to defined subcellular regions and have allowed the study of H_2_O_2_ flux through mitochondrial sub-compartments in the context of a whole, living cell [15,18]. However, we sought a more versatile approach to localization that would allow different sensors to be localized to a sub-mitochondrial compartment without changing the protein expressed there.

Here, we report the use of a small molecule bearing a ligand recognized by a self-labelling protein, in contrast to employing a fluorescent protein-based method. This approach allows us to specifically target an on-off fluorescent H_2_O_2_ reporter to designated sub-mitochondrial compartments, enabling the detection of H_2_O_2_ dynamics in HeLa cells with sub-organellar resolution. In theory, this approach allows localization of any sensor or reporter bearing a ligand for a self-labelling protein. While we focus on ROS dynamics, the reported technology (and indeed our collection of cell lines) could be an extremely useful and versatile tool for the detection of dynamic variations with sub-organellar resolution of any chemical species for which a fluorescent SNAP or Halo ligand can be designed.

Using SNAP-tags to target small fluorescent probes to different sub-cellular regions has become well established in recent years [[22], [23], [24]]. As such, we utilized this approach to specifically target a newly synthesized coumarin-based H_2_O_2_ sensor to as many mitochondrial sub-compartments as possible, in a systematic manner. The SNAP-tags were shown to be correctly positioned in mitochondrial sub-domains as determined using both biochemical localization approaches and optical microscopy approaches, guaranteeing the accuracy of the approach. This chemical biology approach should allow important biological questions to be addressed with a sub-mitochondrial compartment spatial resolution. The advantage of such approaches will be to understand in which mitochondrial sub-compartment certain processes occur, which small molecule species are involved (e.g. different types of mitochondrial ROS) and how these change with time or different metabolic and nutrient conditions. This will provide a more detailed and mechanistically accurate analysis compared to one where a species is detected in mitochondria but without any information about which compartment is involved or whether intramitochondrial changes occur, thus avoiding an oversimplified and potentially misleading view.

We have developed a new coumarin-based arylboronate dye PeroxyCoum, which is not itself fluorescent but generates a fluorescent aminocoumarin dye Coum upon reaction with HOO^−^. We have attached these dyes to a tag that is complementary to SNAP-tag to give a probe that can load them selectively onto SNAP-tag expressed at different sites (Fig. 3). There are two different tags that can be recognized by SNAP-tag, so both the O^6^-benzylguanine and chloropyrimidine tagged coumarins PeroxyCoum BG and CP, together with the tagged fluorophores Coum BG and Coum CP were synthesized for testing in our system (Fig. 2). Congruent with earlier studies on tagged derivatives [22], the chloropyrimidine derivative was found to be more cell permeable than the O^6^-benzylguanine derivative. Given our requirement for the probe to cross multiple membranes to reach the mitochondrial sub-compartments, the chloropyrimidine version PeroxyCoum CP was the logical choice for further experiments.

SNAP-tag was successfully localized to 5 different sites in the mitochondria and to the cytosol in separate cell lines by expressing the SNAP-tag as fusion proteins with differently localized targeting proteins (Fig. 4). The SNAP-tag proteins recognized the CP ligand and the localized Coum was 5- to 10-fold brighter than the PeroxyCoum targeted to the same location showing that a response to H_2_O_2_ would be detectable.

Our approach allowed us to detect H_2_O_2_ flux from the cytosol to the mitochondrial matrix upon exogenous treatment (Fig. 5, Fig. 7). The flux was observed both in cells grown in glucose and those grown in galactose but was more intense for those grown in galactose. The flux we observe is in agreement with two recent studies [38,39] that have shown a directionality of mitochondrial ROS. While there were clear increases in the concentration of H_2_O_2_ in the cytosol and matrix, there was less response from the PeroxyCoum localized in the IMS. A similar apparently low concentration of H_2_O_2_ in IMS followed by a surge in H_2_O_2_ levels within the matrix has been seen in a previous study [15]. Our observations are unlikely to be due to a concentration, or protein expression effect given the high signal from the Coum-SNAP-tag conjugates seen in all subcellular locations (Fig. 4C and Fig. S7). The maximum response from oxidation of PeroxyCoum conjugates by H_2_O_2_ is in the range of a 5–10 fold increase in all sub-compartments as determined from the fluorescence of localized Coum conjugates (Fig. 4). The different redox conditions and presence of endogenous reducing enzymes will likely affect the levels of H_2_O_2_ and different ROS in each compartment, and it is possible that there is rapid scavenging of H_2_O_2_ in the IMS, or diffusion of H_2_O_2_ is faster into the matrix than out of it. However, the most straightforward explanation for the low response in the IMS is the effect of the pH on the HOO^−^ levels in the more acidic IMS. [40,41] Boronate probes are electrophilic and so react with hydroperoxide ions resulting from deprotonation of H_2_O_2_ (pKa = 11.6)^32^. This means that the reaction rate is expected to be about 10-fold faster for a boronate reacting at pH 8 than at pH 7. We confirmed that pH does not affect the fluorescence of Coum (Fig. S9), and demonstrated that PeroxyCoum reacts more slowly with hydrogen peroxide at lower pH, consistent with the lower concentration of HOO^−^ (Fig. S10). Thus, the same amount of hydrogen peroxide in the acidic regions of the intermembrane space would release the dye at less than one tenth of the rate they would release the dye in the matrix. A similar issue is likely to occur with Hyper7. Unlike PeroxyCoum, HyPer7 is nucleophilic in its reduced form and so reacts with H_2_O_2_ itself. Like Coum, its fluorescence is stable to pH. However, the reactivity of the reduced form of HyPer7 would also be expected to decrease at lower pH because thiolates are far more nucleophilic than thiols ^43.^ This affects the thermodynamics for the conversion of the thiol to the disulfide. The disulfide is less favoured at lower pH because the reducing power of a thiol decreases with pH as the fraction of thiolate drops [42,43]. For both types of sensor (PeroxyCoum and Hyper7), it is most likely that the detection of H_2_O_2_ as it is trafficked from the cytosol to the mitochondrial matrix is decreased by the low pH of the IMS sub-compartment it is passing through (Fig. 5B and C). This fully explains why hydrogen peroxide added externally can be detected in the matrix but not in the intermembrane space in both this and the earlier study. [15]

Our results are in good agreement with the results of Hoehne, et al. [15] who have shown that H_2_O_2_ produced within mitochondria, either via Antimycin A treatment or by using a matrix-targeted enzyme d-amino acid oxidase (mtDAO) which produces H_2_O_2_ in response to supplementation with d-alanine [18,44,45], was detectable in the cytosol [15]. This was in contrast to previous data [18], although both groups linked the levels of peroxiredoxins within both the cytosol and the mitochondria to the flux of H_2_O_2_. Using our small molecule-based approach we have been able to confirm many of the findings of both of these studies, but importantly we have enhanced the level of spatial detail available by targeting our probe to multiple regions within the IMS, as well as the cristae lumen. This level of spatial resolution is required to fully appreciate the differences in flux of ROS and the links to subtle internal and structural changes of mitochondria in processes like cristae remodeling and mitochondrial dynamics.

Our experiments using Antimycin A to induce O_2_^•-^ production within the respiratory chain, revealed some interesting results (Fig. 6). Long term exposure led to an increase in fluorescence due to conversion of PeroxyCoum to Coum only in the matrix, suggesting that scavenging mechanisms within the IMS may mitigate H_2_O_2_ production and control its levels within this sub-compartment. On shorter timescales, PeroxyCoum fluorescence was increased in the cristae lumen as well as the matrix, suggesting that there is a time constraint to such H_2_O_2_ scavenging, at least in the cristae lumen, which should be considered a separate compartment to the bulk IMS [46] which is delineated by the boundary IM. Whether antioxidant concentrations within the cristae lumen are increased over longer timescales to counteract this enhanced, potentially localized, oxidative stress is not known, but it seems logical that an increase would occur in the lumen given the location of complex III. Drawing links to the metabolic state of the cells, using galactose media to drive respiration exhibited greater flux of H_2_O_2_ in response to antimycin A treatment. Indeed, we were able to detect H_2_O_2_ in the IMS and even on the outer surface of mitochondria suggesting that it is in fact released under these conditions. No significant increase in dye fluorescence in the cytosol was seen, but this is likely due to the massive volume difference between the mitochondria and the cytosol and variability between experiments due to low TMRE uptake in these cells. TMRE fluorescence was used as a marker for detection of cells and was used for the subsequent quantification of PeroxyCoum signals. While TMRE signals in this cell line were lower than in all other stably expressing cell lines, it is important to note that this did not result in a respiratory phenotype as shown by oxygen consumption assays, suggesting that the cells were not metabolically distinct from any of the other cell lines or indeed wild-type cells. Interestingly, concentrations of H_2_O_2_ within the IMS differed, with higher levels detected close to the OMM as opposed to the vicinity of the IMM. This is an important finding because it provides the first evidence that levels of H_2_O_2_ differ even within the IMS, not just between separate sub-compartments, supporting the concept of finely tuned localized nature of ROS within mitochondria. However, one needs to keep in mind that it may be possible that lower pH conditions close to the IMM reduce the levels of reactive HOO^−^ available to PeroxyCoum. Indeed, this issue may have wider implications for the detection of H_2_O_2_ in this sub-compartment using many different approaches.

Finally, we have demonstrated the binding of two spectrally distinct fluorescent probes to different subcellular and sub-mitochondrial localizations within the same cell using expression of both SNAP and Halo tags. Although we have not expanded our work here to a detailed study incorporating at the same time both SNAP- and Halo targeted probes in all possible sub-compartments, the experiment given in Fig. 8 is providing proof of principle for further studies. Broadly speaking, this paves the way for concomitant, dual (two-color) detection of different ROS within the same cell, using chemical ligands with different reactivity. Based on this proof-of-concept further studies could focus on analyzing the concentrations of various ROS species, or indeed any ion or molecule that can be detected using small chemical probes (one based on a SNAP-tag and the other based on a Halo Tag ligand), within the diverse sub-compartments of mitochondria within the same cell. It should be noted that working with the HaloTag, at least in our hands, was challenging. Many of the constructs tested showed poor mitochondrial localization of HaloTag, and our attempts to generate stable cell lines were unsuccessful, presumably due to toxicity problems due to HaloTag overexpression. We were however able to transiently express constructs for cytosolic HaloTag and an IMS targeted HaloTag attached to the N-terminal sequence of AIFM1, allowing its labeling with a chloroalkane-tagged fluorophore within the SNAP-tag-expressing cell lines.

In conclusion, we present a chemical biology approach to target small molecule fluorescent indicators to all mitochondrial sub-compartments for highly spatially accurate data on H_2_O_2_ flux in living mammalian cells. We have shown that fluorescent dyes targeted appropriately can be used to detect H_2_O_2_ fluxes from inside and from outside the mitochondria and that increased amounts of H_2_O_2_ are produced and can be released from mitochondria under specific metabolic conditions. The possibilities offered by this approach are very broad and could empower studies for example on human disease mutations thought to be linked to metabolic alterations and ROS production, or probing in more detail the role of mitochondrial ROS in ageing cells. Importantly, we show that this technology can be applied for the targeting of multiple small molecule probes to different sites within the same mitochondria, paving the way for future studies (using multiplexing formats) to examine the concomitant sub-mitochondrial dynamics of different key molecules involved in signalling and stress response pathways in cells.

## 5 Conclusion

Mitochondrial ROS dynamics are of critical importance to cellular function and have implications for many human diseases. The measurement of these molecules with sub-organellar resolution is of vital importance to understand how they are transported throughout the organelle and the cell as a whole. Here we present a small molecule approach allowing the specific imaging and quantitative detection of H_2_O_2_ at unprecedented spatial resolution within mitochondria. Harnessing this approach, we discovered that cells grown in galactose media (favoring respiration) display increased H_2_O_2_ flux compared to glucose media (favoring glycolysis), with marked differences between sub-mitochondrial compartments. In addition, we demonstrated that SNAP-tag and HaloTag could be positioned in different compartments of the same mitochondria and labelled with spectrally distinct fluorophores so that they could be observed concomitantly. This paves the way for the *in-situ* measurement of any chemical species, at multiple locations within the same cell, for which a functional probe can be designed.

## CRediT authorship contribution statement

**Ross Eaglesfield:** Writing – review & editing, Writing – original draft, Visualization, Validation, Methodology, Investigation, Formal analysis, Data curation, Conceptualization. **Erika Fernandez-Vizarra:** Writing – review & editing, Writing – original draft, Visualization, Validation, Methodology, Investigation, Formal analysis, Data curation. **Erik Lacko:** Methodology, Investigation. **Stuart T. Caldwell:** Writing – review & editing, Visualization, Validation, Methodology, Investigation, Formal analysis, Data curation. **Nikki L. Sloan:** Methodology, Investigation. **Daniel Siciarz:** Formal analysis, Data curation. **Richard C. Hartley:** Writing – review & editing, Writing – original draft, Visualization, Validation, Supervision, Methodology, Funding acquisition, Formal analysis, Data curation, Conceptualization. **Kostas Tokatlidis:** Writing – review & editing, Writing – original draft, Visualization, Validation, Supervision, Project administration, Methodology, Investigation, Funding acquisition, Formal analysis, Data curation, Conceptualization.

## Funding

This work was supported by the UK Research and Innovation-Biotechnology and Biological Sciences Research Council (UKRI-BBSRC grants BB/T003804/1, BB/R009031/1, BB/X511948/1). STC was funded in part by a 10.13039/100010269Wellcome Trust Investigator award to RCH (220257/B/20/Z). For the purpose of open access, the author has applied a CC BY public copyright licence to any Author Accepted Manuscript version arising from this submission.

## Declaration of competing interest

The authors declare no conflicts of interests.

## Data Availability

Data will be made available on request.
