# Supplementary material for: Sub-organellar mitochondrial hydrogen peroxide observed using a SNAP tag targeted coumarin-based fluorescent reporter
Source: Redox Biol. 2025 Jan 20;80:103502. doi: 10.1016/j.redox.2025.103502 (PMC11802384; doi:10.1016/j.redox.2025.103502)
Supplement: Multimedia component 1 [file mmc1.docx]

**SUPPLEMENTAL INFORMATION**

**Sub-organellar mitochondrial hydrogen peroxide observed using a SNAP tag targeted coumarin-based fluorescent reporter**

Ross Eaglesfield^1,3^, Erika Fernandez-Vizarra^1,4^, Erik Lacko^1^, Stuart T. Caldwell^2^, Nikki L. Sloan^2^, Daniel Siciarz^1^, Richard C. Hartley^2*^ and Kostas Tokatlidis^1*^

1. School of Molecular Biosciences, University of Glasgow, UK, G12 8QQ

2. School of Chemistry, University of Glasgow, UK, G12 8QQ

3.Present address: National Renewable Energy Laboratory, Golden, CO, USA

4. Present address: Department of Biochemistry and Molecular and Cellular Biology, Faculty of Health and Sport Sciences, University of Zaragoza, Spain 22002

*Corresponding authors:

[Kostas.Tokatlidis@glasgow.ac.uk](mailto:Kostas.Tokatlidis@glasgow.ac.uk) and Richard.Hartley@glasgow.ac.uk

@KostasTokatlid

**Lead contact:** Further information and requests for resources and reagents should be directed to and will be fulfilled by the lead contact **Kostas Tokatlidis** (Kostas.tokatlidis@glasgow.ac.uk)

**(Ten supplemental figures S1-S10)**

**Figure S1.** **The numbering and lettering used to report assignment of protons in ^1^H NMR methods section (e.g. H-4, H-5', H_A_).**

**Figure S2.** **Fluorescence analysis of chemical synthesis compounds**

(A) Fluorescence emission of a 1 μM solution of compounds **7** and **9** in pH 7.4 PBS buffer (Ex 410 nm) showing an ~ 20 fold increase in emission between the caged and free amino coumarin.

(B) UV/Visible absorption of a 10 μM solution of compounds **7** and **9** in pH 7.4 PBS buffer.

**Figure S3. Pseudo first order kinetic of the uncaging of 9.** Caged coumarin **9** (10 μM in pH 7.4 PBS buffer) was added to a 200 μM solution of H_2_O_2_ at 37 °C. The reaction was monitored by the increase in absorption at 405 nm.


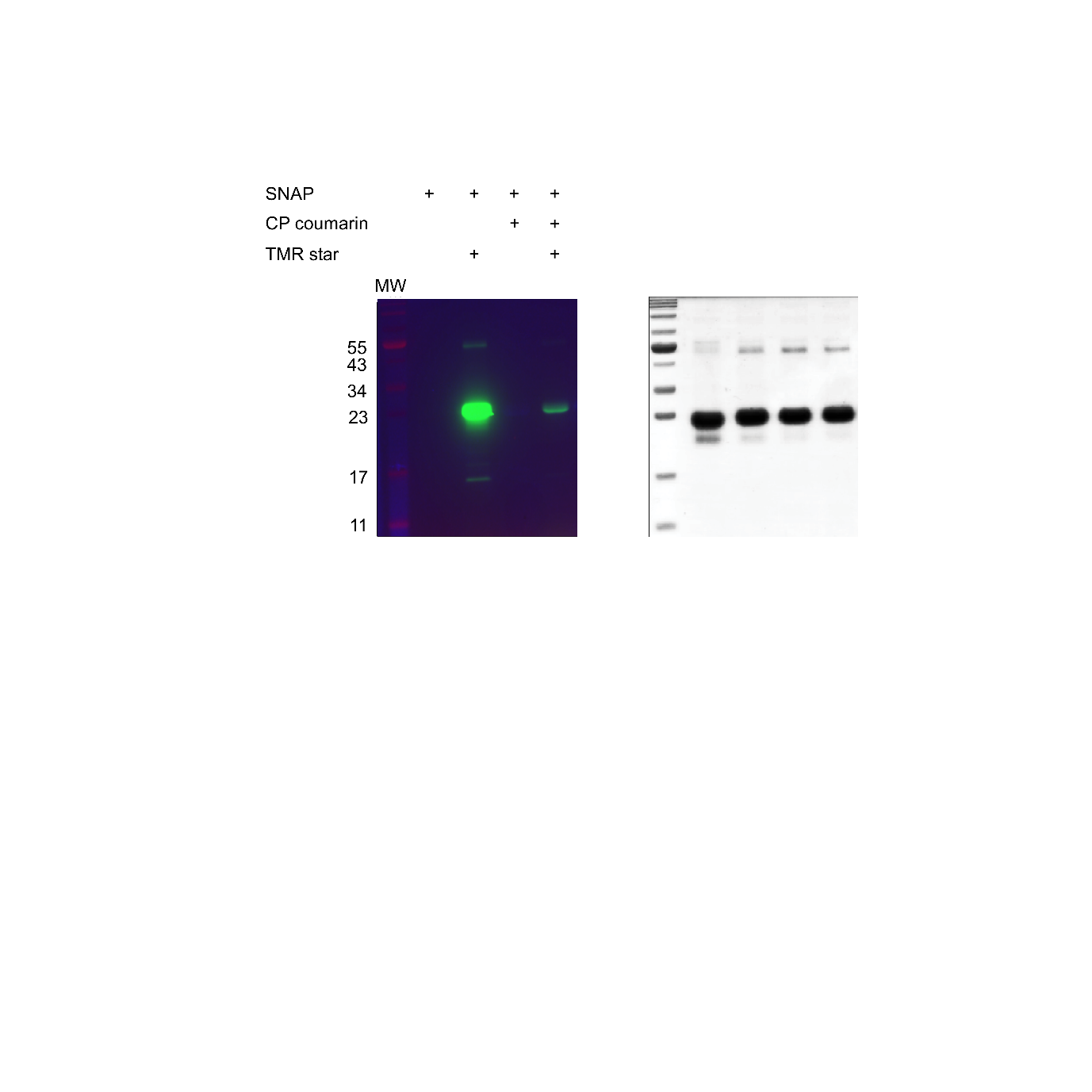


**Figure S4. In gel analysis of CP coumarin (Coum CP) binding to SNAP tag**. The left panel is a fluorescent competition assay showing that CP coumarin successfully competes for SNAP binding with the fluorescent SNAP ligand TMR star. The fluorescence of TMR star was detected using a Azure Biosystems C400 Imaging system equipped with RGB LEDS filters at a wavelength of 554 nm. The coumarin fluorescence is outside this range but the successful binding was determined by the decrease in fluorescence when both ligands were added to the SNAP protein (fourth lane). The right panel shows the corresponding Coomassie stained gel.


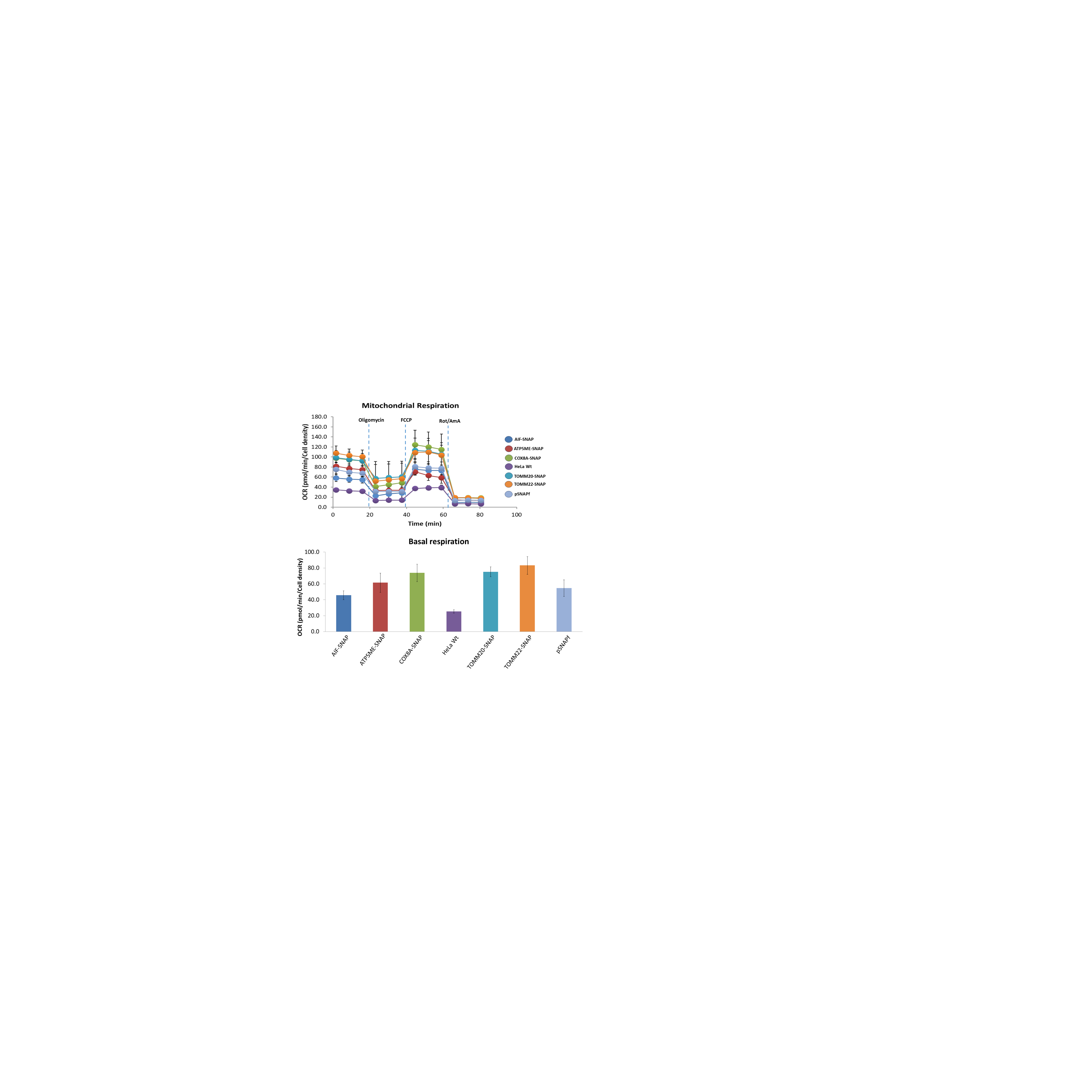


**Figure S5. Oxygen consumption rate assay**. All cell lines stably expressing the SNAP-tagged proteins targeted to the different mitochondrial compartments, and WT HeLa cells were subjected to the MitoStress test using a Seahorse XF Pro Analyzer instrument (Agilent). The graph shows the mean ± SD of sextuplicate experiments. Cells were dosed according to the manufacturer’s instructions with 1.5 μM oligomycin, 2 μM FCCP and 0.5 μM of both rotenone and antimycin A. Data was normalised to cell density quantified by NucBlue (Invitrogen) nuclear stain fluorescence using a BMG Labtech POLARstar OMEGA plate reader according to the manufacturer’s (Agilent) application note for normalisation: [**https://www.bmglabtech.com/en/application-notes/normalisation-of-seahorse-xfe96-metabolic-assays-to-cell-number-with-hoechst-stain/#:~:text=This%20labelling%20method%20produces%20a,Wave%E2%80%9D%20software%20to%20normalise%20results**](https://www.bmglabtech.com/en/application-notes/normalisation-of-seahorse-xfe96-metabolic-assays-to-cell-number-with-hoechst-stain/#:~:text=This%20labelling%20method%20produces%20a,Wave%E2%80%9D%20software%20to%20normalise%20results.). The relevant publication in the literature is Tao et al 2024 ^30^

**
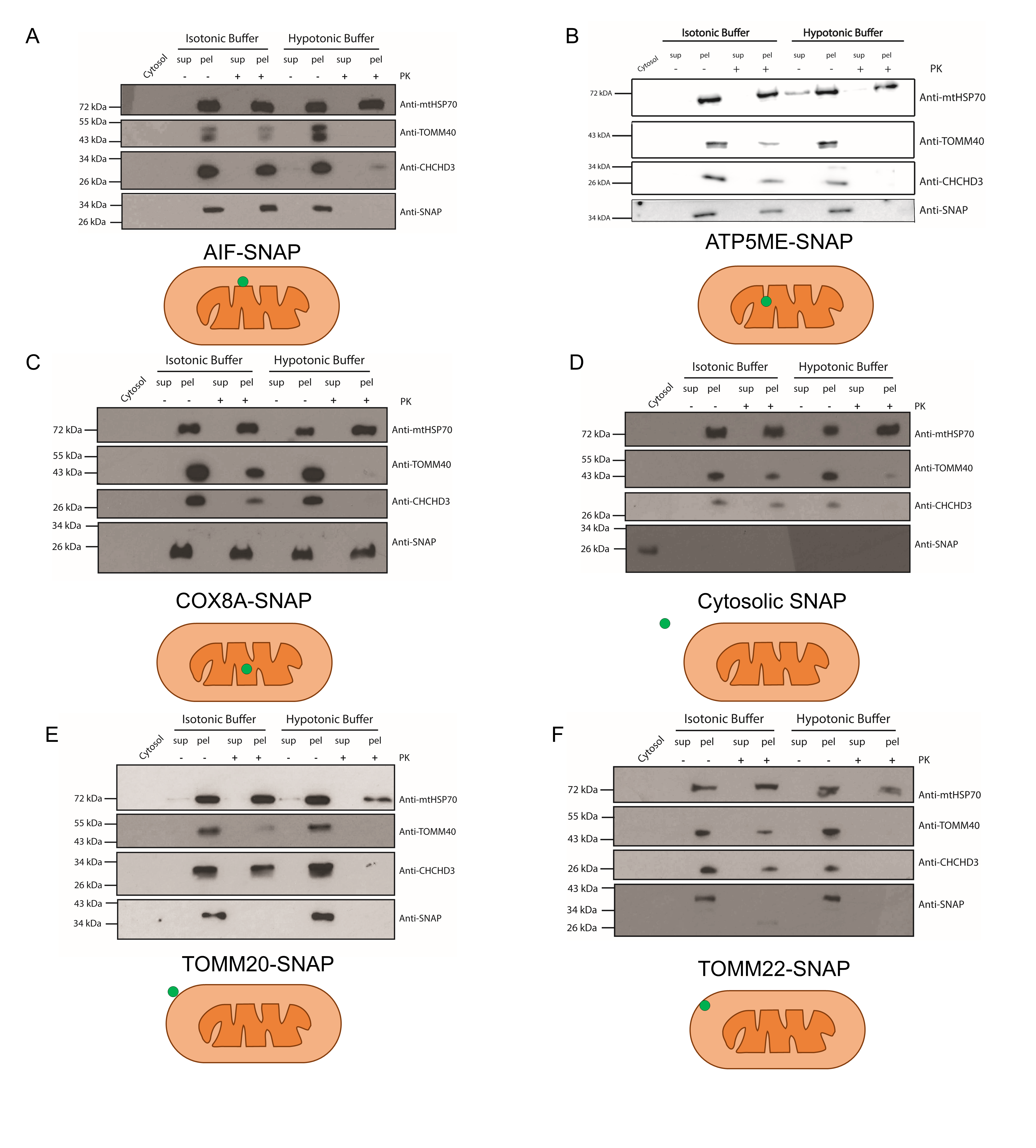
**

**Figure S6. Biochemical localisation of SNAP tags in purified mitochondria and mitoplasts from HeLa cells.** Mitochondria were purified from HeLa cells using established protocols (see Methods) and cytosolic fractions were taken during the purification process. Mitochondria were treated with either isotonic (to maintain outer membrane integrity) or hypotonic (to generate mitoplasts) buffers both with and without proteinase K treatment. Supernatant (sup) and pellet (pel) fractions were then taken and all fractions were run on 12 % SDS-PAGE gels (sup fractions were precipitated with 10 % TCA prior to loading). Resulting gels were transferred onto nitrocellulose membranes before probing with antibodies against mtHSP70, TOMM40, CHCHD3 and SNAP.

(A)AIF-SNAP - IMS. (B) ATP5ME-SNAP - cristae lumen. (C) COX8A-SNAP - cytosol. (E) TOMM20-SNAP - OMM facing the cytosol. (F) TOMM22-SNAP - OMM facing the IMS.


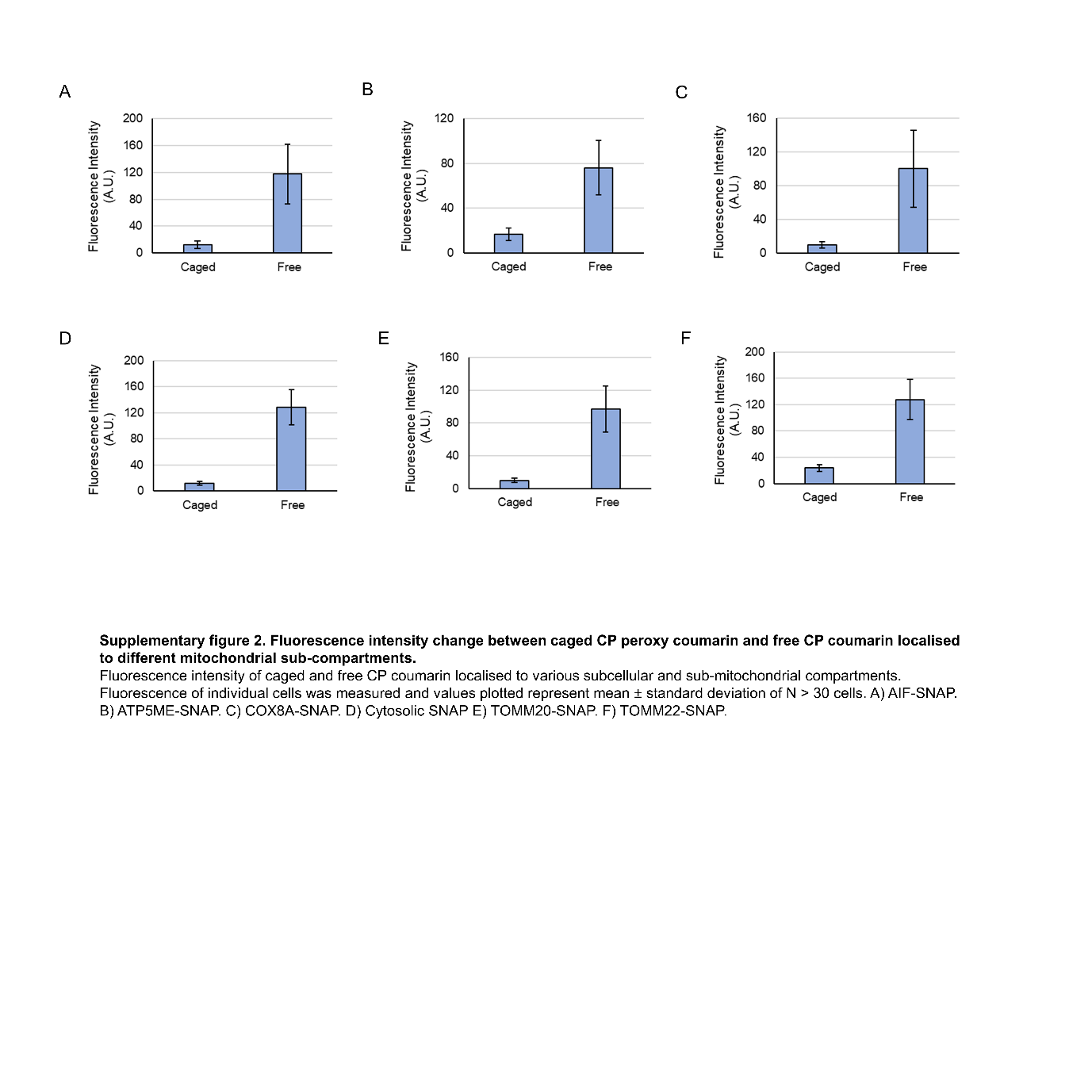


**Figure S7. Fluorescence intensity change between caged CP peroxy coumarin and free CP coumarin localized to different mitochondrial sub-compartments.** Fluorescence intensity of caged and free CP coumarin localized to various subcellular and sub-mitochondrial compartments. Fluorescence of individual cells was measured and values plotted represent mean +/- standard deviation of N > 30 cells. (A) AIF-SNAP. (B)ATP5ME-SNAP. (C) COX8A-SNAP. (D) Cytosolic SNAP. (E)TOMM20-SNAP. (F)TOMM22-SNAP.


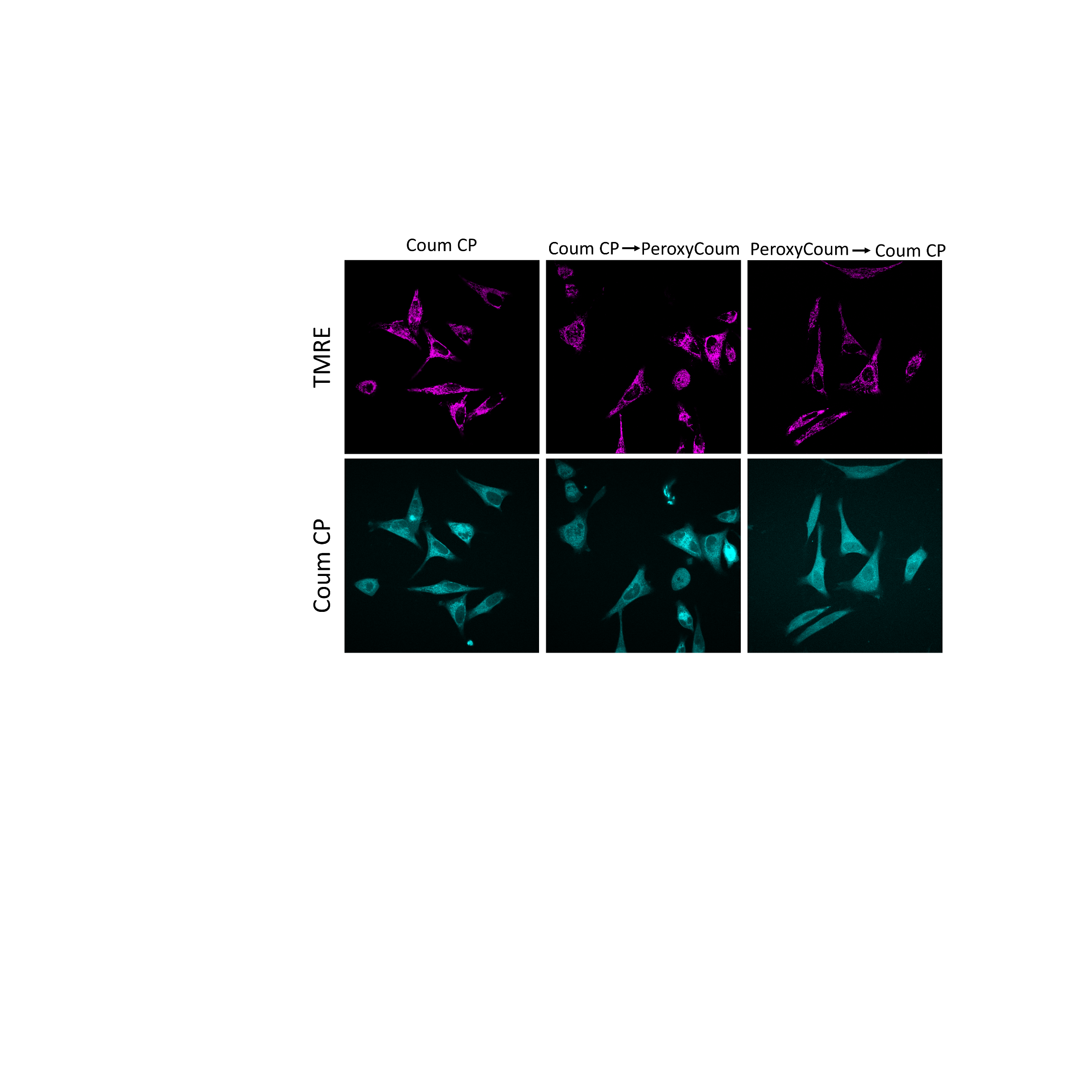


**Figure S8. Competition of Coum CP and PeroxyCoum CP for binding to matrix-localized SNAP-tag.**

Representative confocal microscopy images of Coum CP binding to mitochondrial matrix targeted SNAP-tag, where PeroxyCoum CP is added post, or prior to incubation with Coum CP. The COX8a-SNAP cell line is used for localisation to the matrix, and TMRE as a mitochondrial stain.


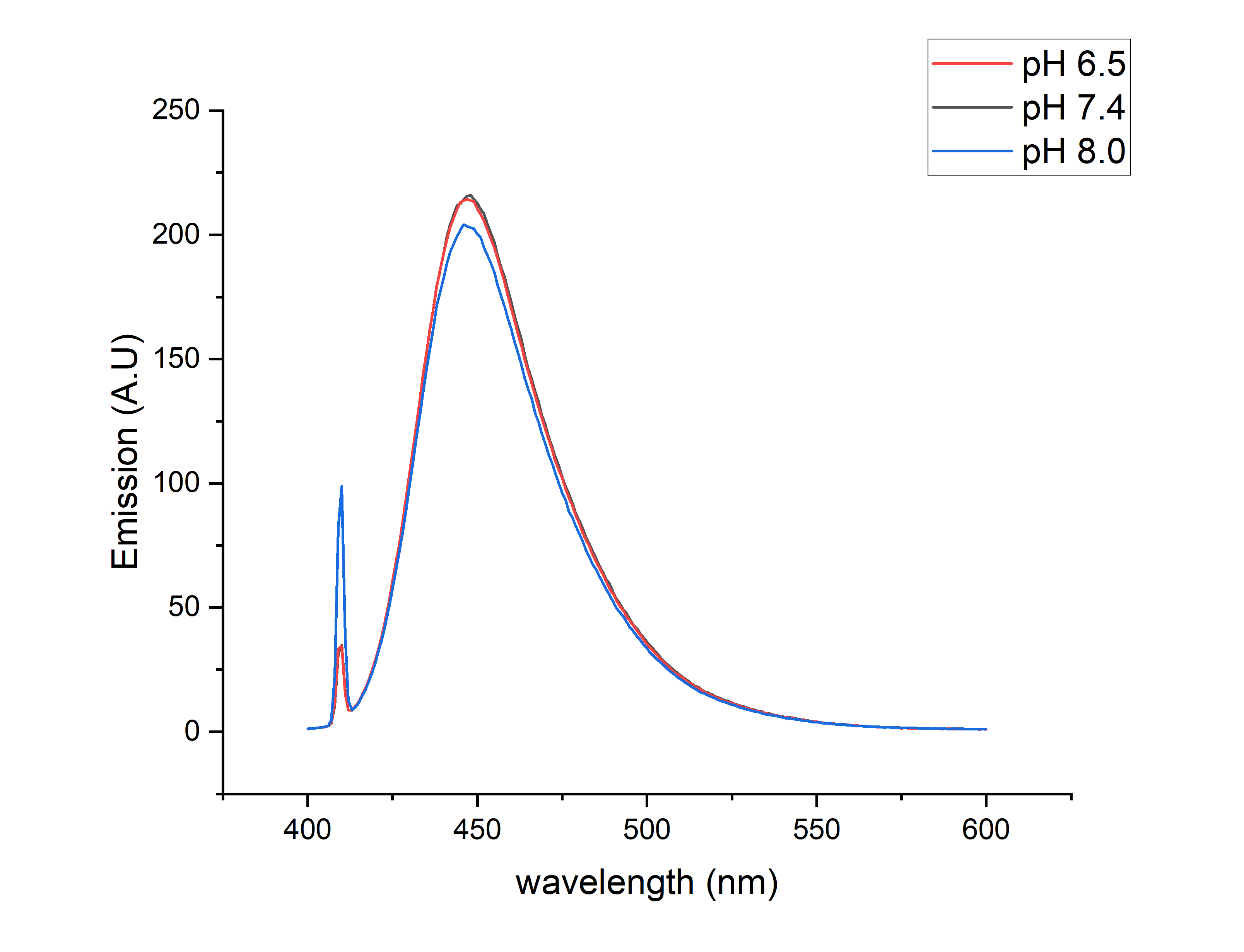


**Figure S9**. Fluorescence emission of a 1 μM solution of coumarin **7** in pH 6.5 (PBS), 7.4 (PBS) or 8.0 (HEPES) buffer. Each spectra are each an average of three recordings from three separate dilutions of the same stock solution for each pH. Excitation 410 nm


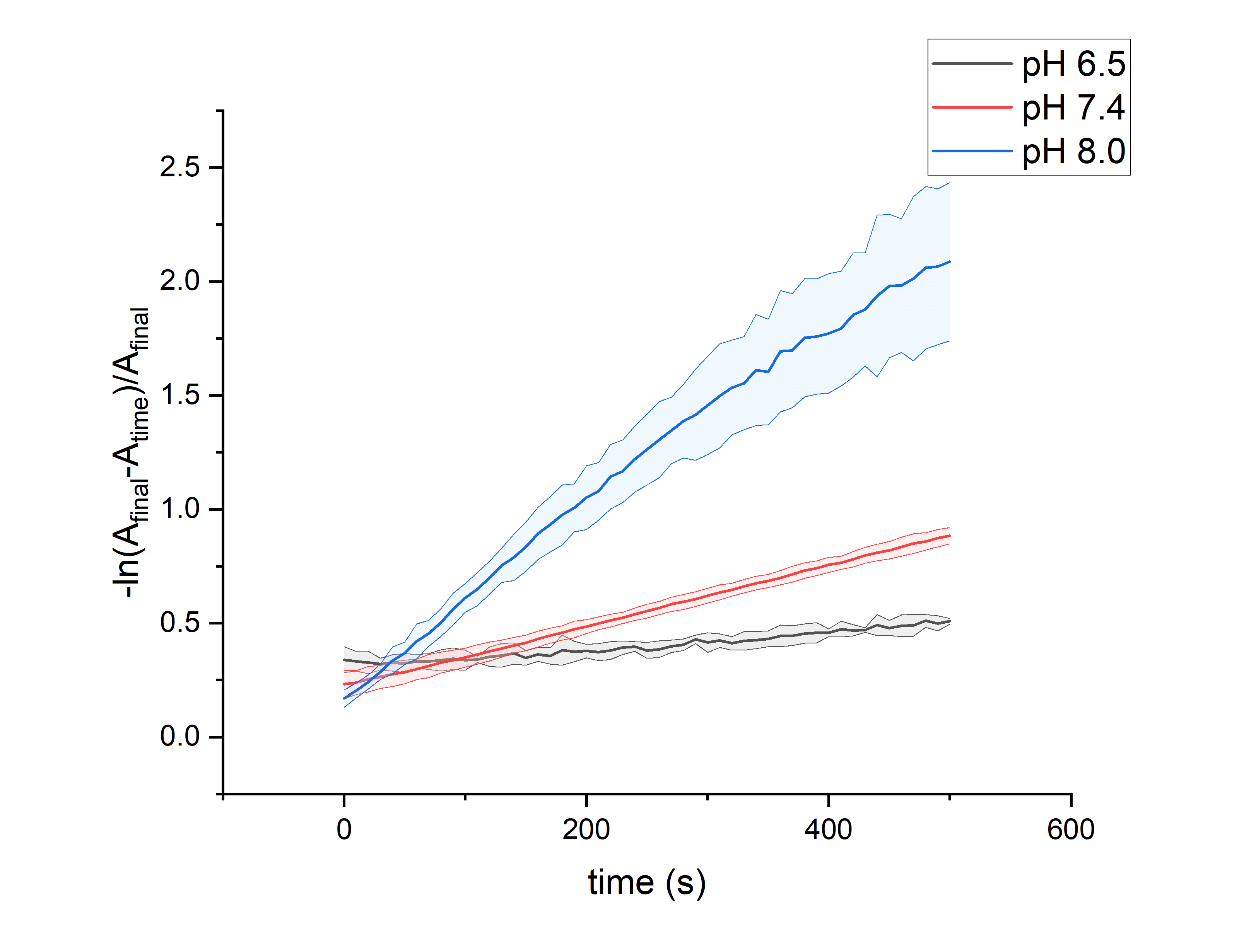


**Figure S10. Lagergren plots of** **pseudo first order kinetics of reaction of arylboronate 9 with H_2_O_2_.** Hydrogen peroxide (final concentration 400 μM) was added to a solution of arylboronate **9** (10 μM in either pH 6.5 (PBS), 7.4 (PBS) or 8.0 (HEPES) buffer to determine initial rates. The data are presented as an average of three separate runs for each pH, and the shaded areas show the standard deviation. The linear regions of each line were selected to calculate pseudo first order kinetics: 200 s for pH 8.0, 500 s for other pHs. Pseudo first order rate constants are k’ = 3.81 ×10^- 4^ (±0.26) s^-1^ (pH 6.5 PBS), 1.33 ×10^-3^ (±0.13) s^-1^ (pH 7.4 PBS) and 4.54 ×10^-3^ (±0.53) s^-1^ (pH 8.0 HEPES). Calculated second order rate constants are 0.952 (±0.065) M^-1^s^-1^ (pH 6.5), 3.32 (±0.33) M^-1^s^-1^ (pH 7.4), 11.3 (±1.3) M^-1^s^-1^ (pH 8.0).
